# Polydopamine Nanodots Ameliorate Inflammatory Bowel Disease by Restoring Redox Homeostasis and Intestinal Microenvironment

**DOI:** 10.1002/advs.202508674

**Published:** 2025-08-23

**Authors:** Zhen Ding, Xingfu Bao, Ying Zhao, Lin Bai, Jinming Zhang, Chengjing Xu, Tianyan Chen, Shuang Dai, Yufu Liu, Min Hu, Meng Qin, Zhen Liu

**Affiliations:** ^1^ Jilin Provincial Key Laboratory of Tooth Development and Bone Remodeling School and Hospital of Stomatology Jilin University Changchun 130021 China; ^2^ Beijing Advanced Innovation Center for Soft Matter Science and Engineering College of Life Science and Technology Beijing University of Chemical Technology Beijing 100029 China; ^3^ Department of Lung Cancer Center Center for Preclinical Safety Evaluation of Drugs State Key Laboratory of Biotherapy West China Hospital Sichuan University Chengdu 610041 China; ^4^ Histology and Imaging Platform West China Hospital Sichuan University Chengdu 610041 China

**Keywords:** inflammatory bowel disease, oral formulation, polydopamine nanodots, targeted therapy

## Abstract

Efficient elimination of intestinal reactive oxygen species (ROS) and accelerated alleviation of proinflammatory microenvironment are considered as a direct approach for the management of inflammatory bowel disease (IBD). Although promising, current treatments focusing on the intestinal inflammation resolution are still unsatisfactory and far from their practical applications. To address these limitations, novel polydopamine nanodots (PDA NDs) are rationally designed and developed as an efficient oral formulation for the targeted IBD treatment by regulating intestinal oxidative stress and proinflammatory microenvironment. Because of their intrinsic chemical structure, PDA NDs not only show high stability in the harsh gastrointestinal environment but also allow the precise targeting accumulation around the inflamed colon, suggesting a significant improvement in the oral therapeutic efficacy. After establishing murine models with different degrees of IBDs, oral administration of PDA NDs to diseased mice can well eliminate excessive oxidative stress, alleviate intestinal inflammation, ameliorate intestinal barrier integrity, as well as rebalance gut microflora. Furthermore, orally delivered PDA NDs exhibit high safety and extremely low toxicity in murine model, providing enough confidence for the further development of targeted IBD treatments. Overall, the current study has proposed a safe, facile, and highly efficient therapeutic strategy for the comprehensive management of IBDs.

## Graphical abstract

A novel melanin‐based therapeutic composed of ultrasmall polydopamine nanodots is employed as an efficient oral formulation for the targeted treatment of inflammatory bowel disease. The potential therapeutic mechanism of polydopamine nanodots mainly involves the elimination of excessive oxidative stress, the reduction of ROS‐mediated proinflammatory response, the amelioration of intestinal barrier function, as well as the restoration of normal gut microbiota.

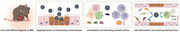

## 1 Introduction

As a group of gastrointestinal disorders instigated by the interactions between environmental, genetic, infectious, and immune factors, inflammatory bowel disease (IBD) has imposed a substantial burden on both patients and society.^[^ ^1^ ^]^ Although the etiology and pathogenesis of IBD are not well understood, emerging evidences suggest that IBD is highly associated with the dysfunction of intestinal barrier and disturbance in the gut microflora.^[^ ^2^ ^]^ Furthermore, a severe immune response induced by the enrichment of positively charged proteins and elevated levels of reactive oxygen species (ROS) has been demonstrated around the inflamed colon.^[^ ^3^ ^]^ Due to the complexity and diversity of inflammatory microenvironment in the intestinal lesions and the existence of patients who do not response to a single anti‐inflammatory treatment, developing efficient therapies for IBDs with different degrees is still a challenge.^[^ ^4^ ^]^ More importantly, current clinical treatments relying on the long‐term use of small‐molecule drugs or immunosuppressants usually lead to serious off‐target systemic side effects and complications including immunological response, antibiotic resistance, as well as unwanted organ damage.^[^ ^5^ ^]^ Given the limitations of current small‐molecule therapeutics or biologics, there is an urgent need for the development of innovative therapeutic strategies that can efficiently eliminate multiple danger elements and exhibit reduced systemic toxicity.

As a well‐known endogenous biopolymer in most living organisms including humans with great biocompatibility and biodegradability, melanin can serve as a central regulator in many physiological processes.^[^ ^6^ ^]^ Arising from their fascinating functions including near‐infrared absorbance, chelating capacity, antibiotic effect, and free radical scavenging ability, natural or artificial melanin and their derivatives have been well developed as contrast agents, photothermal agents, chelating agents, antibacterial agents, and antioxidants for biomedical applications.^[^ ^7^ ^]^ Because of their similar chemical structure to natural polyphenols, melanin and its derivatives exhibit admirable immunomodulation effect and can be utilized in the efficient neuroendocrine regulation and sleep assistance.^[^ ^8^ ^]^ As a series of common artificial melanin, polydopamines and their derivatives have been reported to be able to reduce immune overactivation in inflamed tissue and promote tissue‐resident memory differentiation of T cells.^[^ ^9^ ^]^ In that case, we envision that melanin and their derivatives may exhibit more promising in scavenging multiple ROS and regulating proinflammatory microenvironment around lesions. Although promising, recent studies including ours have primarily focused on the use of single function of melanin and their derivatives in the treatment of inflammatory diseases including IBD, thus hindering their applicability to differences in diseases and neglecting their potential therapeutic outcome.^[^ ^10^ ^]^ More importantly, there are still tremendous challenges in the design of ideal melanin‐based oral therapeutics, although some therapies have been developed for the targeted IBD treatment. For instance, some essential barriers remain to be addressed, such as biocompatibility restricted by the non‐clinical chemicals, low bioavailability limited by the uncontrollable synthesis, and poor therapeutic efficacy owing to the lack of targeting capability.**^[^** ^11^ **^]^** Therefore, it is imperative to explore a simple yet efficient strategy to develop a patient‐friendly melanin‐based oral therapeutic and improve its targeting ability of inflamed lesions, maximizing its functions including elimination of intestinal ROS and alleviation of proinflammatory microenvironment.

**Figure 1:**
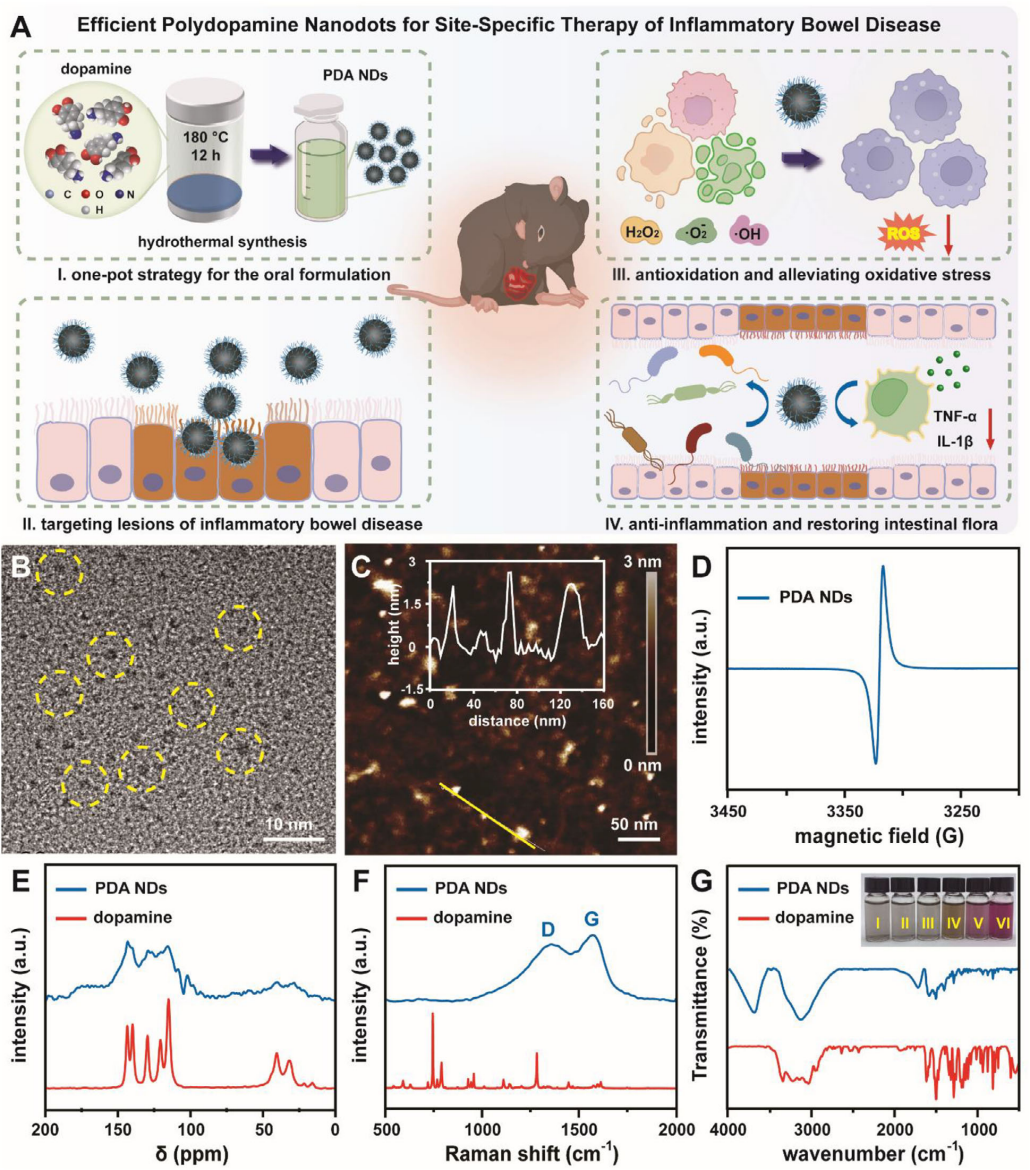
Schematic illustration of the synthesis of PDA NDs with antioxidant activity and their application in the targeted treatment of IBDs with admirable repair function of intestinal microenvironment (A). TEM image (B), AFM image (C), and ESR spectrum (D) of PDA NDs. Inset of (C): height profile of PDA NDs. ^13^C NMR spectra (E), Raman spectra (F), and FT‐IR spectra (G) of PDA NDs and dopamine. Inset of (G): photo of PDA NDs dispersed in different media including water, 0.9% NaCl, PBS, FBS, RPMI‐1640, and DMEM.

To tackle these challenges, we for the first time revealed that a novel melanin‐based therapeutic composed with ultrasmall polydopamine nanodots (PDA NDs) could be employed as an efficient oral formulation for the targeted IBD treatment (**Figure** **1**A). These negatively charged PDA NDs with broad‐spectrum antioxidant activity and superior stability in gastrointestinal tract could be naturally targeted toward the intestinal lesions through the strong electrostatic interactions. According to our current design, oral administration of PDA NDs to diseased mice with different degrees of IBDs exhibited a stabilizing effect on the intestinal microenvironment by reducing excessive oxidative stress, alleviating intestinal inflammation, improving damaged intestinal barrier function, as well as balancing the intestinal microflora. Notably, the therapeutic effect of PDA NDs was superior to that of classical polydopamine nanoparticles with an average diameter of 160 nm. Evidenced by the microbiome analysis and transcriptomics analysis, oral administration of PDA NDs could rebalance gut microflora by increasing the abundance of microbial communities and the quantity of probiotics, as well as regulate the expression of ROS and inflammation‐related genes during the targeted therapy. In addition, a long‐term toxicity study indicated the high biosafety and biocompatibility of PDA NDs. Taken together, our well‐designed melanin‐based targeted platform with high therapeutic outcomes held a great promise for the comprehensive management of gastrointestinal disorders including IBDs with different degrees.

## 2 Results and Discussion

### 2.1 Preparation and Characterization of PDA NDs

As illustrated in Figure S1A (Supporting Information), PDA NDs were rationally synthesized using a facile one‐pot hydrothermal approach. Transmission electron microscope (TEM) image revealed that the resultant PDA NDs held an average diameter of 3 nm with a dot‐like morphology (Figure 1B). Meanwhile, atomic force microscope (AFM) image demonstrated that these well‐developed PDA NDs were homogeneous with a mean topographic height of 3 nm (Figure 1C). In order to well understand the controllability for the synthesis of PDA NDs, time‐dependent experiments were designed and performed. Photos of dispersions containing products with different colors indicated that different degrees of carbonization occurred during the hydrothermal synthesis (Figure S1B, Supporting Information). Based on the TEM images, a reaction period shorter than 6 h failed to generate ideal product while a reaction period of 12 h could yield a similar product with that of 24 h (Figure S1C,D, Supporting Information). From the viewpoint of time saving and practical applications, 12 h was selected as the optimal reaction period in the typical synthesis. Some previous studies demonstrated that the self‐polymerization or oxidation of dopamine could lead to the generation of melanin‐like products with stable π‐electron free radicals. Prior to confirming the melanin‐like chemical structure of PDA NDs, classical PDA nanoparticles (PDA NPs) with an average diameter of 160 nm were prepared via a classical self‐polymerization approach.^[^ ^12^ ^]^ Figure S2 (Supporting Information) revealed the detailed morphological information and essential characterization of classical PDA NPs. Evidenced by the electron spin resonance (ESR) technique, our PDA NDs exhibited a single‐peak ESR spectrum with a g‐factor around 2 (Figure 1D). Similar to the classical PDA NPs, irregular cross‐linked polymer networks containing mixed bonding arrangements and radicals localized around quinone residues could be easily found in our PDA NDs. More importantly, our PDA NDs also had a sharper absorbance peak, which indicated that they held a narrower range of bonding arrangements than natural melanin. These findings could suggest the successful synthesis of PDA NDs with a well‐defined melanin‐like chemical structure. According to the ^13^C nuclear magnetic resonance (NMR) spectra of PDA NDs and dopamine, a sharp peak at δ = 129 ppm referred to the sp^2^ carbon in graphite, a peak at δ = 143 ppm corresponded to the carbon in the phenolic hydroxyl group, as well as other peaks represented the carbon in the indole and pyrrole units (δ = 102 ppm), aliphatic carbon (δ = 28, 40 ppm), and carbonyl carbon (δ = 172 ppm), demonstrating the occurrence of carbonization and self‐polymerization during the synthesis of PDA NDs (Figure 1E). Notably, our PDA NDs had a similar ^13^C NMR spectrum to that of classical PDA NPs. Complementary to the ESR and ^13^C NMR results, Raman spectrum of PDA NDs, which was highly consistent with those of natural melanin and classical PDA NPs, provided additional evidence for the successful synthesis of novel melanin‐like particles (Figure 1F). According to the fourier transform infrared (FT‐IR) spectra, the absorbance peak at 3200 cm^−1^ in PDA NDs could be attributed to the presence of methylene while the absorbance peak at 1700 cm^−1^ could be assigned to the stretching vibration of aromatic ring and the bending ring vibration of N─H (Figure 1G). Meanwhile, the observed C─OH stretching vibration and ─OH bending vibration at 1500–1000 cm^−1^ further demonstrated the presence of abundant hydrophilic groups on the surface of PDA NDs. Obviously, the active groups in PDA NDs had much higher recognition in FT‐IR spectrum compared with that of PDA NPs due to the difference in diameter between them. X‐ray photoelectron spectroscopy (XPS) was then utilized to analyze the chemical composition of PDA NDs. As shown in Figure S3 (Supporting Information), the peak at 284.6 eV of the C1s spectrum was generated by the σ bond between C elements in indole framework while the peak at 286.0 eV could be attributed to the C─O bond in o‐phenol and the C─N bond in indole ring.^[^ ^13^ ^]^ The peak at 286.8 eV was generated by C─N bond while the peak at 288.7 eV could be ascribed to the π bond between C elements in benzene ring or C═O in quinone group. Evidenced by the O1s and N1s spectra, there were more active groups in PDA NDs including C─OH peak at 532.6 eV and C═O peak at 531.0 eV in the O1s spectrum, as well as N─H peak at 401.5 eV and C─N peak at 399.9 eV in the N1s spectrum. In addition, the zeta potentials of both PDA NDs and classical PDA NPs were negative, suggesting their high biosafety (Figure S4, Supporting Information). Last but not least, our PDA NDs exhibited admirable colloidal stability in various physiological solutions. Collectively, these results indicated the sucessful synthesis of melanin‐like PDA NDs and their high potential for in vivo applications.

### 2.2 Free Radical Scavenging Efficacy of PDA NDs

**Figure 2:**
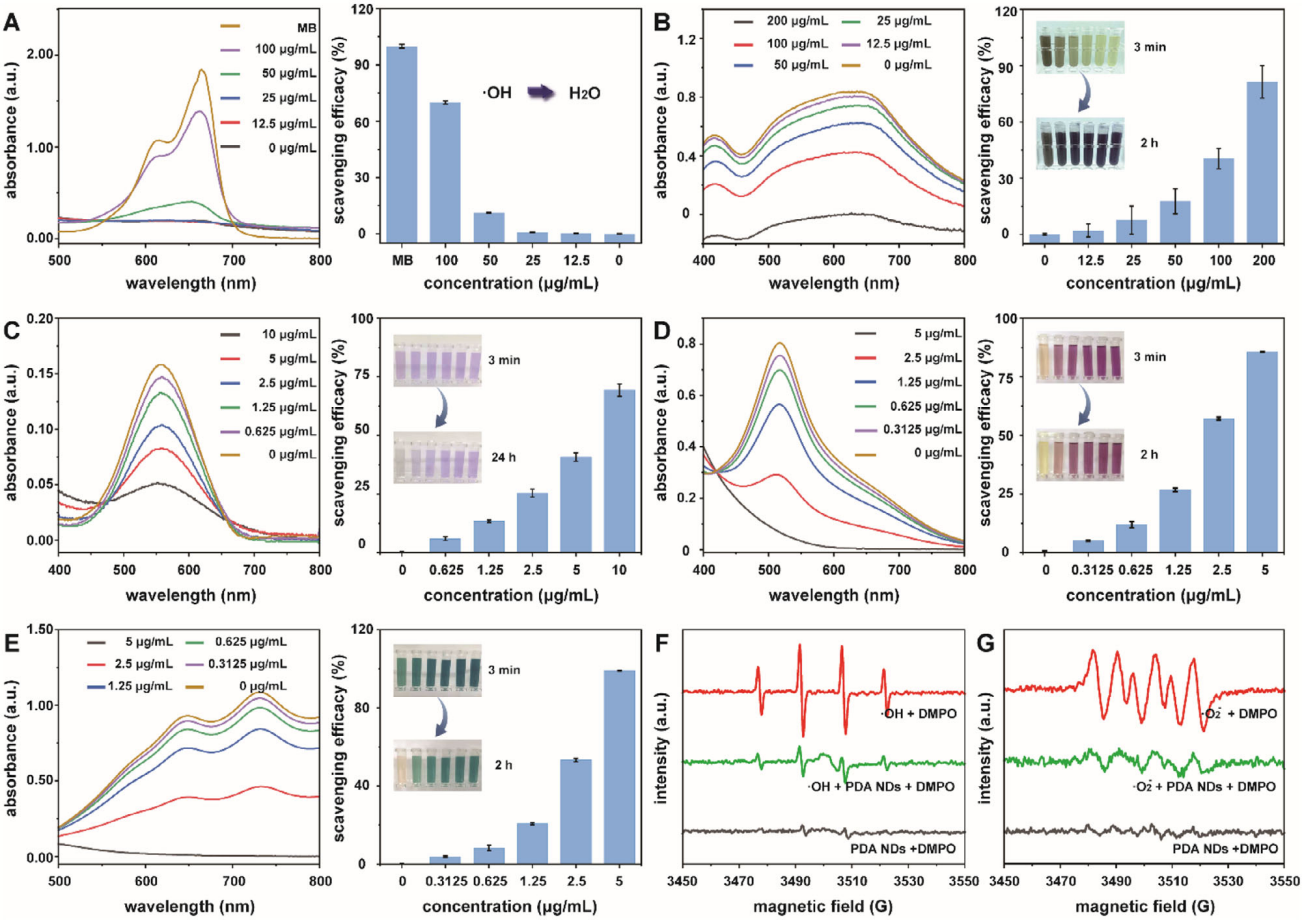
UV–vis absorption spectra and the corresponding scavenging percentages of PDA NDs toward different ROS and their derivatives including ·OH (A), ·O_2_^−^ (B), PTIO· (C), DPPH· (D), and ABTS^+^· (E). Scavenging efficacy of PDA NDs toward free radicals including ·OH (F) and ·O_2_^−^ (G) recorded by ESR spectra. Data in (A–E) are presented as mean ± SD (*n* = 3).

After understanding the structural properties of these well‐developed PDA NDs, we further explored their scavenging efficacy against diverse free radicals.^[^ ^14^ ^]^ Based on our current design, ·OH, ·O_2_^−^, PTIO·, DPPH·, and ABTS^+^· were selected and utilized as typical free radicals to verify the broad‐spectrum antioxidant activity of PDA NDs. Initially, we investigated the scavenging performance of PDA NDs toward ·OH with ultrashort life and strong oxidation activity. In common, ·OH could be generated from H_2_O_2_ via Fenton reaction in the presence of Fe^2+^. Upon the treatment of ·OH, methylene blue could convert into its oxidized form with the color fading and a reduction in the characteristic absorbance at 665 nm. The addition of PDA NDs could efficiently eliminate ·OH and reduce the oxidation of methylene blue, leading to the retention of its color. **Figure** **2**A revealed that PDA NDs featured a rapid scavenging rate toward ·OH and an admirable antioxidant activity in a concentration‐dependent manner. In detail, more than 70% ·OH could be eliminated by PDA NDs with a concentration of 100 µg mL^−1^. Subsequently, the scavenging performance of PDA NDs toward ·O_2_^−^ was explored. In principle, L‐methionine could serve as an electron donor for riboflavin. Upon the white light irradiation, riboflavin together with surrounding oxygen could generate ·O_2_^−^. Nitrotetrazolium blue chloride could react with the resultant ·O_2_^−^ to form blue formazan with a characteristic absorbance at 560 nm. By detecting the residual of formazan, we could assess the scavenging performance of PDA NDs toward ·O_2_^−^. As shown in Figure 2B, the absorbance values of formazan decreased sharply with the addition of PDA NDs and the color of dispersion gradually changed. Meanwhile, the scavenging efficacy of PDA NDs toward ·O_2_^−^ exhibited an approximately linear relationship with their concentration. Moreover, PTIO· was selected as a stable oxygen‐centered free radical and utilized to confirm the ROS‐scavenging performance of PDA NDs. As expected, the elimination of PTIO· using PDA NDs could induce a significant reduction in its characteristic absorption at 557 nm (Figure 2C). When the concentration of PDA NDs increased from 0.625 to 10 µg mL^−1^, the clearance percentage of PTIO· increased from 5.94% to 60.84%. As the main derivatives of ROS, reactive nitrogen species (RNS) also played an essential role during the physiological and pathological processes of living organisms. The scavenging performance of PDA NDs against typical RNS including DPPH· and ABTS^+^· was provided in Figure 2D,E. Our results suggested the remarkable scavenging performance of PDA NDs toward both DPPH· and ABTS^+^·, which also followed a concentration‐dependent manner. Considering the high activity and short lifetime of ·OH and ·O_2_^−^, ESR technique was then utilized to assess the ROS scavenging efficacy of PDA NDs. Using 5,5‐dimethyl‐1‐pyrroline N‐oxide (DMPO) as a typical capture reagent, the signals of both DMPO+·OH and DMPO+·O_2_^−^ decreased sharply with the addition of PDA NDs while PDA NDs themselves did not exhibit obvious ESR signals in the presence of DMPO (Figure 2F,G). Moreover, DPPH· was utilized as a typical free radical to validate the antioxidant efficacy among dopamine, PDA NPs and PDA NDs. As shown in Figure S5 (Supporting Information), PDA NPs and PDA NDs held a similar antioxidant property compared with that of dopamine, and the ability of PDA NDs to eliminate DPPH· was much higher than that of PDA NPs. Thus, these exciting results highlighted the excellent scavenging performance of PDA NDs toward various ROS and RNS owing to their ultrasmall size‐induced large specific surface area and abundant antioxidant active groups.

### 2.3 Biosafety Evaluation of PDA NDs

**Figure 3:**
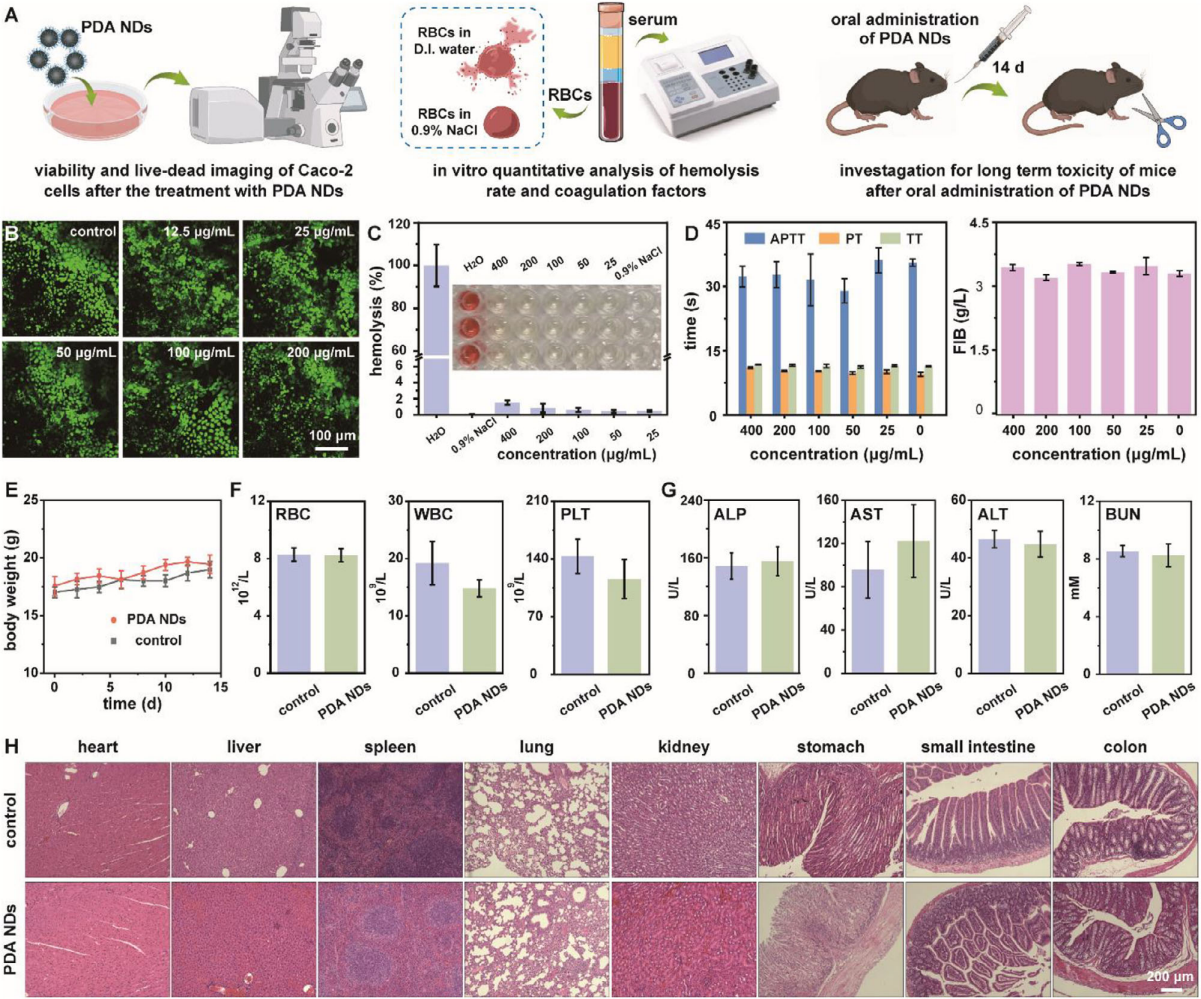
Schematic illustration for the toxicity assessment of PDA NDs (A). Concentration‐dependent live/dead staining images of Caco‐2 cells treated with PDA NDs (B). Hemolysis (C) and coagulation time (D) in the presence of PDA NDs. Changes of mouse body weight (E), hematological index (F), blood biochemical index (G), and histopathological analysis according to H&E staining (H) after oral administration of PDA NDs. Data in (C), (D), (F), and (G) are presented as mean ± SD (*n* = 3). Data in (E) are presented as mean ± SD (*n* = 5).

Prior to their use in the IBD treatment both in vitro and in vivo, a series of experiments were designed and performed for the biosafety evaluation of PDA NDs.^[^ ^15^ ^]^ **Figure** **3**A illustrated the detailed toxicity assessment of PDA NDs. According to the MTT‐based colorimetric assay, PDA NDs held no obvious cytotoxicity toward Caco‐2 cells at any co‐incubation concentration (Figure S6A, Supporting Information). Meanwhile, PDA NDs held negligible effects on the membrane integrity of Caco‐2 cells even at a concentration of 200 µg mL^−1^ based on the lactate dehydrogenase (LDH) leakage assay (Figure S6B, Supporting Information). Moreover, live/dead staining results indicated that PDA NDs could not affect the morphology and the visible viability of Caco‐2 cells (Figure 3B). Besides that, PDA NDs exhibited no obvious cytotoxicity toward RAW264.7 cells according to the above‐mentioned assays (Figure S6C–E, Supporting Information). Hemolysis assay and blood coagulation assay were critical for evaluating the hemocompatibility of any newly developed nanomedicine. Quantitatively, all the hemolysis rates were lower than 2%, implying that no hemolysis occurred in the presence of PDA NDs (Figure 3C; Table S1, Supporting Information). Prothrombin time (PT), thrombin time (TT), activated partial thromboplastin time (APTT), and fibrinogen (FIB) were then measured to verify the effects of PDA NDs on the coagulation function. As expected, all the data were within the reference values, indicating that PDA NDs did not lead to severe effects on different blood coagulation time (Figure 3D; Table S2, Supporting Information). To validate the in vivo toxicity of PDA NDs, we recorded the change in body weight, as well as collected blood, urine, and main exposed organs from mice. The body weight change curve of mice after oral administration of PDA NDs was consistent with that of non‐administered mice (Figure 3E). Meanwhile, indexes including hematology, blood biochemicals, and urine of mice in the PDA NDs group held similar results with those of non‐administered mice (Figure 3F,G; Table S3, Supporting Information). Typical hematology indexes included white blood cell (WBC), red blood cell (RBC), and platelet (PLT) while main blood biochemical indexes involved alkaline phosphatase (ALP), aspartate transaminase (AST), alanine aminotransferase (ALT), and blood urea nitrogen (BUN). Figure 3H revealed that no distinguishable abnormalities or inflammatory lesions were found in the above two groups according to the histological analysis of the main organs. Last but not least, oral administration of PDA NPs did not exhibit obvious in vivo toxicity, which could be utilized as the essential control for further investigation (Figure S7, Supporting Information). Thus, these results suggested the high biosafety of these well‐prepared PDA NDs after oral administration.

### 2.4 Stability and Mucoadhesive Property of PDA NDs

**Figure 4:**
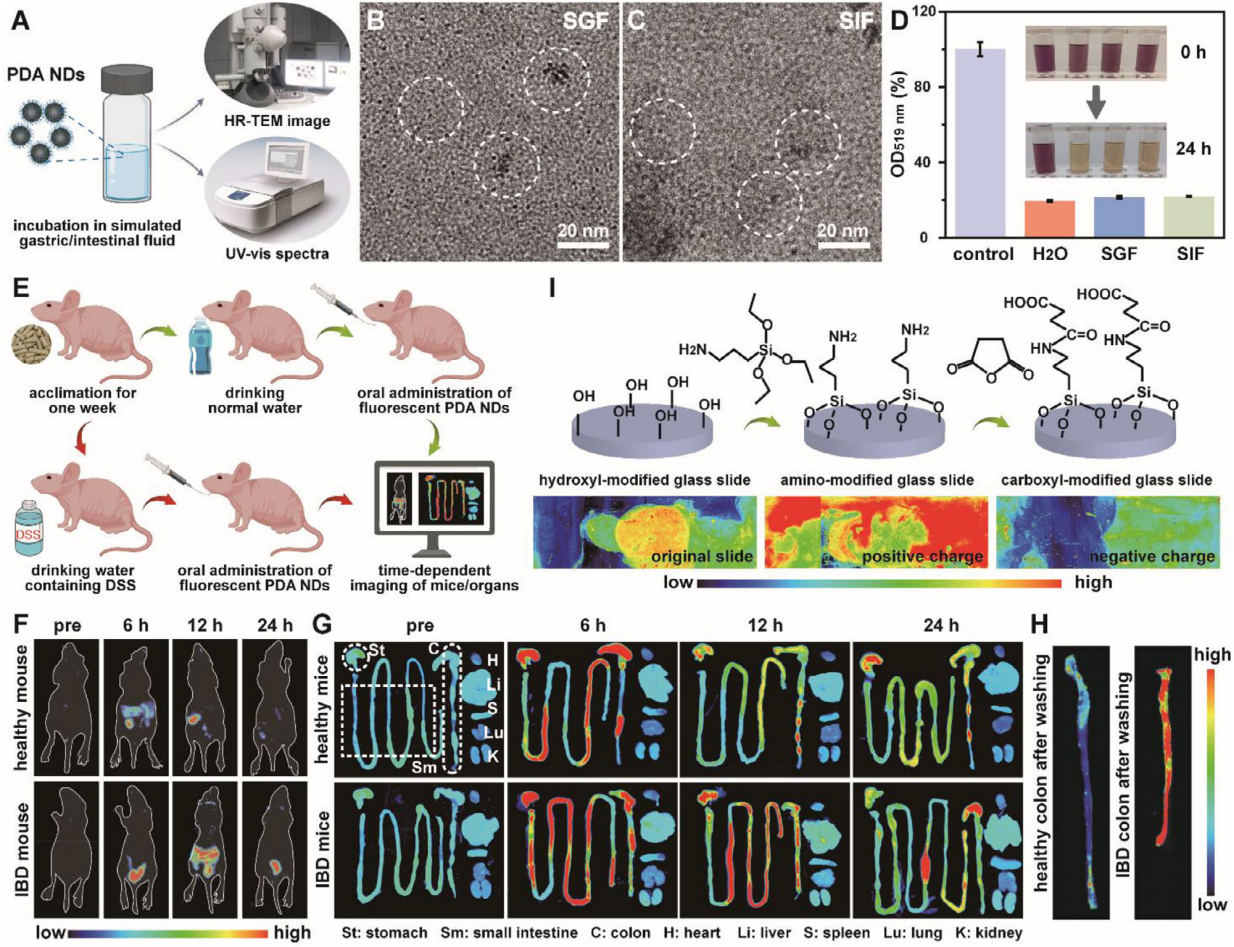
Schematic illustration for the evaluation of PDA NDs after co‐incubation with SGF or SIF (A). TEM images of PDA NDs treated with SGF (B) and SIF (C). Scavenging efficacy of PDA NDs toward DPPH· after different treatments (D). Schematic illustration for fluorescence imaging of healthy and IBD mice (E). Time‐dependent in vivo (F) and ex vivo (G) fluorescence imaging of healthy and IBD mice after oral administration of Cy5.5‐modified PDA NDs. Fluorescence imaging of colons from healthy and IBD mice after repeated washing (H). The above IBD mice were developed by oral administration of DSS with a concentration of 3%. The preparation of glass slides with different charges and fluorescence imaging of the interaction between slides and Cy5.5‐modified PDA NDs (I). Data in (D) are presented as mean ± SD (*n* = 3).

Stability of any newly developed oral preparation was considered as the precondition for further determination of intestinal colonization and targeting ability.^[^ ^16^ ^]^ **Figure** **4**A illustrated the detailed approaches for the evaluation of PDA NDs after co‐incubation with simulated gastric fluid (SGF) or simulated intestinal fluid (SIF). According to the TEM images, PDA NDs could well remain their dot‐like morphology without any size change after the co‐incubation (Figure 4B,C). As shown in Figure S8A (Supporting Information), the dispersion containing PDA NDs had a broad absorption across the visible light range in a concentration‐dependent manner. Figure S8B (Supporting Information) revealed that there were no significant changes in the absorption values of PDA NDs before and after the co‐incubation. Meanwhile, we explored the time‐dependent zeta potential changes of PDA NDs in SGF and SIF, respectively. As expected, PDA NDs could well maintain their initial potentials over time, implying that long‐term incubation in SGF or SIF could not lead to the chemical structure change of PDA NDs (Figure S8C,D, Supporting Information). Moreover, the antioxidant activity of PDA NDs before and after the co‐incubation with SGF or SIF was studied using DPPH·‐assisted colorimetric assay. As shown in Figure 4D, PDA NDs with or without treatment held a similar DPPH· scavenging rate, suggesting the co‐incubation with SGF or SIF could not alter the antioxidant activity of PDA NDs.

Following confirmation of the excellent physiological stability of PDA NDs, we further evaluated their bio‐distribution in healthy and IBD mice, respectively (Figure 4E). Prior to the in vivo experiments, fluorescent PDA NDs, defined as Cy5.5‐modified PDA NDs, were initially prepared (Figure S9A, Supporting Information). TEM image revealed that fluorescent PDA NDs had a similar diameter with that of original PDA NDs (Figure S9B, Supporting Information). Fluorescent spectra of both Cy5.5‐modified PDA NDs and Cy5.5 held the same emission peak, indicating the successful synthesis of fluorescent PDA NDs (Figure S9C, Supporting Information). Zeta potential analysis indicated that Cy5.5‐modified PDA NDs were negatively charged particles, promising their biomedical applications (Figure S9D, Supporting Information). To evaluate the mucoadhesive property, healthy and IBD nude mice were orally administrated with fluorescent PDA NDs and observed under an in vivo imaging system. At first, IBD mice were achieved after oral administration of dextran sulfate sodium salt (DSS). According to the in vivo and ex vivo imaging, stronger fluorescence signals could be easily found in the gastrointestinal tract of IBD mice compared to healthy mice (Figure 4F,G). Significantly, there were nearly no fluorescence signals in the small intestine and colon of healthy mice 12 h after the oral administration of PDA NDs. In contrast, strong fluorescence signals were observed in the colon of IBD mice even 24 h after the oral administration of PDA NDs. More importantly, the alteration of fluorescence intensity of colons from healthy and IBD mice after washing implied that PDA NDs exhibited enhanced mucoadhesive property in IBD mice, promising their potential in the targeted IBD treatment (Figure 4H). Furthermore, we developed a mild IBD model in mice by decreasing the daily oral dose and duration. Compared to the healthy mice, stronger fluorescence signals could be easily detected in the gastrointestinal tract of mice with mild IBD (Figure S9E, Supporting Information). Along with the passage of time, fluorescence signals in the small intestine and colon of healthy mice after the oral administration of PDA NDs decreased sharply. However, strong fluorescence signals could be clearly observed in the colon of mice with mild IBD even 24 h after the oral administration of PDA NDs. All these results implied that oral administration of DSS at different dosages could develop IBD model in mice with different degrees. More importantly, the affected area might involve a portion of the small intestine except for the colon.^[^ ^2^, ^16^ ^]^ Some previous studies demonstrated that the excessive accumulation of transferrin proteins, antimicrobial peptides, and bactericidal/permeability‐increasing protein at the damaged sites could result in the formation of inflamed intestinal epithelia with positive charges. To verify the applicability of the above‐mentioned hypothesis in explaining our experimental results, synthetic glass slides with different kinds of charges were prepared to simulate healthy and inflamed intestinal epithelia (Figure 4I). After co‐incubation of fluorescent PDA NDs on carboxylated slide, aminated slide, and uncoated slide, the adsorbed PDA NDs on different slides were analyzed with the help of fluorescence imaging system. As expected, aminated slide retained the highest fluoresence signal compared with carboxylated slide and uncoated slide. The preferential adsorption of PDA NDs onto the aminated slide could be ascribed to the strong electrostatic interactions between them. Together, these results indicated that our well‐developed negatively charged PDA NDs exhibited intrinsic targeting ability toward the inflamed sites in IBDs.

### 2.5 In Vitro Antioxidant Activity of PDA NDs

**Figure 5:**
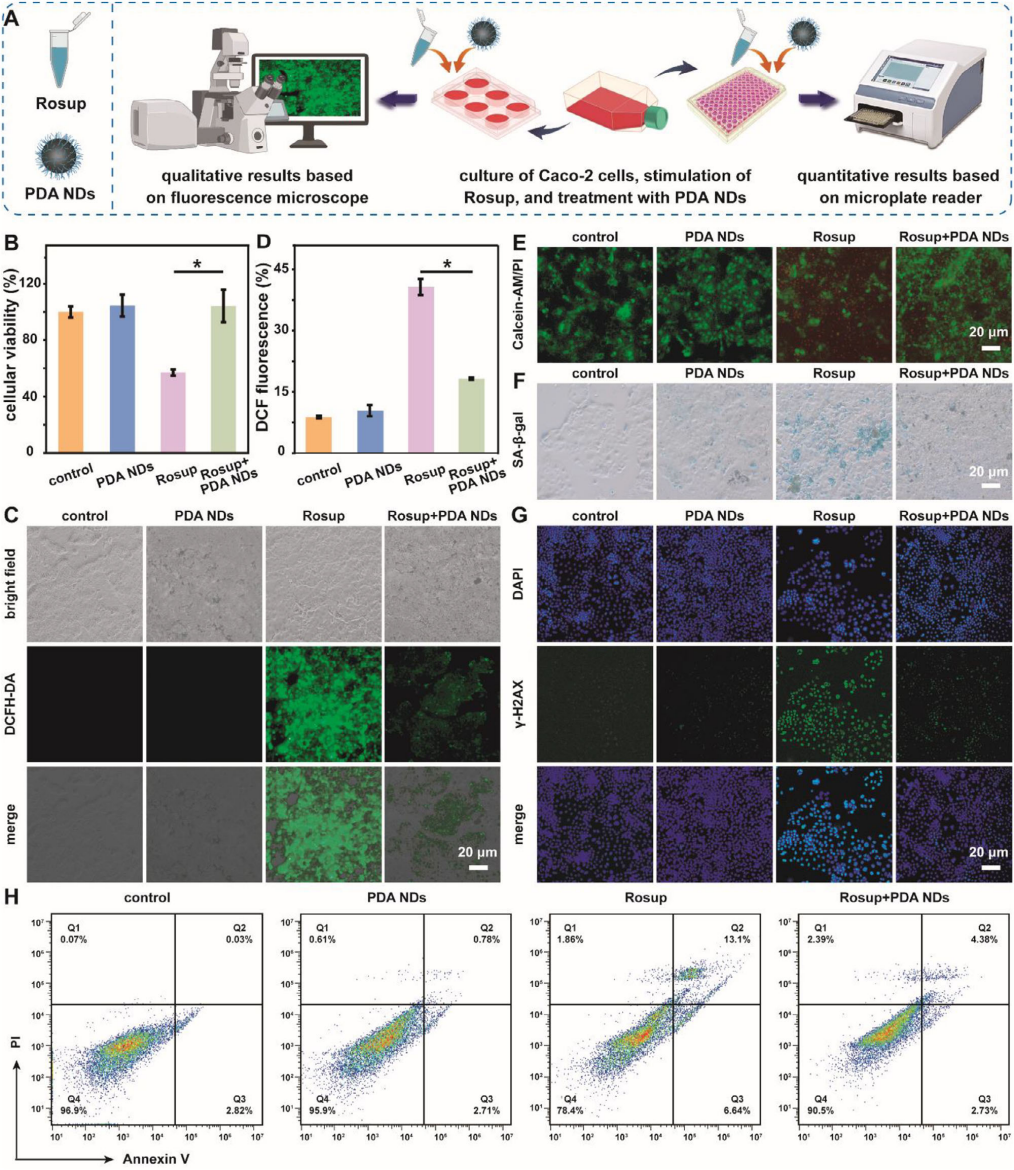
Schematic illustration for the assessment of intracellular antioxidant therapy using PDA NDs (A). Protective effects of PDA NDs on Rosup‐treated Caco‐2 cells (B). Fluorescence images of Caco‐2 cells after different treatments indicated by DCFH‐DA (C) and the corresponding fluorescence intensity obtained by flow cytometry (D). Live/dead staining (E), SA‐β‐gal staining (F), and γ‐H2AX staining (G) of Caco‐2 cells receiving different treatments. Anti‐apoptotic performance of PDA NDs according to flow cytometry (H). Data in (B) and (D) are presented as mean ± SD (*n* = 3). Statistical significance is calculated using unpaired *t*‐test. *p <0.05.

Because of the remarkable free radicals scavenging ability of PDA NDs in tube experiments, we further investigated their feasibility of scavenging intracellular overproduced ROS. The endocytosis performances of Caco‐2 cells toward different nanoparticles were well explored at first. As shown in Figure S10 (Supporting Information), both PDA NPs and PDA NDs could be internalized by Caco‐2 cells. It was worth noting that the endocytosis effect of Caco‐2 cells toward PDA NDs was much higher than those toward PDA NPs, which could be attributed to the differences in cellular internalization induced by the size effect of nanoparticles. The above results therefore indicated that PDA NDs could efficiently eliminate intracellular ROS after their internalization by Caco‐2 cells. Subsequently, we explored the ROS scavenging ability of PDA NDs at cellular level. **Figure** **5**A illustrated the detailed experimental protocols of PDA NDs‐assisted antioxidant therapy. As a typical positive reagent for the generation of ROS, Rosup was co‐incubated with Caco‐2 cells to induce intracellular oxidative stress. According to the MTT assay results, the viability of Rosup‐treated Caco‐2 cells increased with the addition of PDA NDs, indicating their excellent cytoprotecting ability (Figure 5B). It was worth noting that PDA NDs‐treated cells could recover to more normal viability compared to the PDA NPs‐treated ones at the same dose (Figure S11, Supporting Information). Subsequently, the levels of intracellular ROS were monitored using 2′,7′‐dichlorofluorescein diacetate (DCFH‐DA), a common fluorescent probe, through fluorescence microscope imaging and flow cytometry. Figure 5C revealed that Caco‐2 cells treated with Rosup held strong fluorescence intensity, suggesting the high level of intracellular ROS. With the addition of PDA NDs, the intracellular ROS level decreased as expected. Significantly, PDA NDs could reduce the intracellular ROS level in a concentration‐dependent manner by comparing the fluorescence intensity (Figure S12A, Supporting Information). Quantitative analysis of intracellular ROS levels based on flow cytometry re‐confirmed our results of fluorescence imaging, implying that PDA NDs could efficiently eliminate intracellular ROS (Figure 5D; Figure S12B, Supporting Information). It was well known that the accumulation of excessive ROS might disrupt cellular redox homeostasis, induce serious DNA damage and inflammatory response, as well as trigger unwanted cellular senescence and death. As shown in Figure 5E, the living and dead state of Caco‐2 cells treated with PDA NDs was similar to that of the control group as observed under a fluorescence microscope. Compared to the normal Caco‐2 cells, the green fluorescence from the living cells in the Rosup group significantly reduced, while the red fluorescence from the dead cells greatly increased. With the addition of PDA NDs, the number of dead cells in the Rosup+PDA NDs group dramatically decreased, indicating that PDA NDs could efficiently scavenge ROS and alleviate intracellular oxidative stress. Previous studies demonstrated that ROS‐damaged cells usually exhibited a significant senescent phenotype with high levels of senescence‐associated 𝛽‐galactosidase (SA‐𝛽‐gal) activity.^[^ ^17^ ^]^ Thus, the SA‐𝛽‐gal assay was utilized to ensure whether PDA NDs were beneficial to alleviate cellular senescence. As expected, the expression of SA‐𝛽‐gal in the Rosup‐treated Caco‐2 cells was highly inhibited after the addition of PDA NDs, suggesting the admirable antioxidant effect of PDA NDs (Figure 5F; Figure S13A, Supporting Information). Since excessive ROS could lead to the breaks of double‐stranded DNA, we then examined the Rosup‐induced DNA damage using the 𝛾‐H2AX staining assay (Figure 5G; Figure S13B, Supporting Information).^[^ ^18^ ^]^ Nearly no green fluorescence signals were detected in the untreated Caco‐2 cells and PDA NDs‐treated cells while the Rosup‐treated cells held obvious green fluorescence with a high signal‐to‐noise (S/N) ratio. However, with the addition of PDA NDs, the intensity of green fluorescence in the Rosup+PDA NDs group decreased sharply, suggesting that PDA NDs could efficiently protect DNA from Rosup‐induced damage. Last but not least, flow cytometry was utilized to confirm the anti‐apoptotic effect of PDA NDs in the presence of Rosup. Figure 5H reveals that Caco‐2 cells could undergo obvious apoptosis in the group of Rosup, whereas the apoptotic cells decreased after the treatment with PDA NDs. These results indicated that the addition of PDA NDs could extremely relieve the unwanted cell apoptosis and necrosis caused by Rosup. Accordingly, well‐prepared PDA NDs could be considered as a high‐performance nano‐sized modulator as they could efficiently mitigate the intracellular dyshomeostasis and dysfunction caused by oxidative stress.

### 2.6 Treatment of DSS‐Induced Acute Colitis

**Figure 6:**
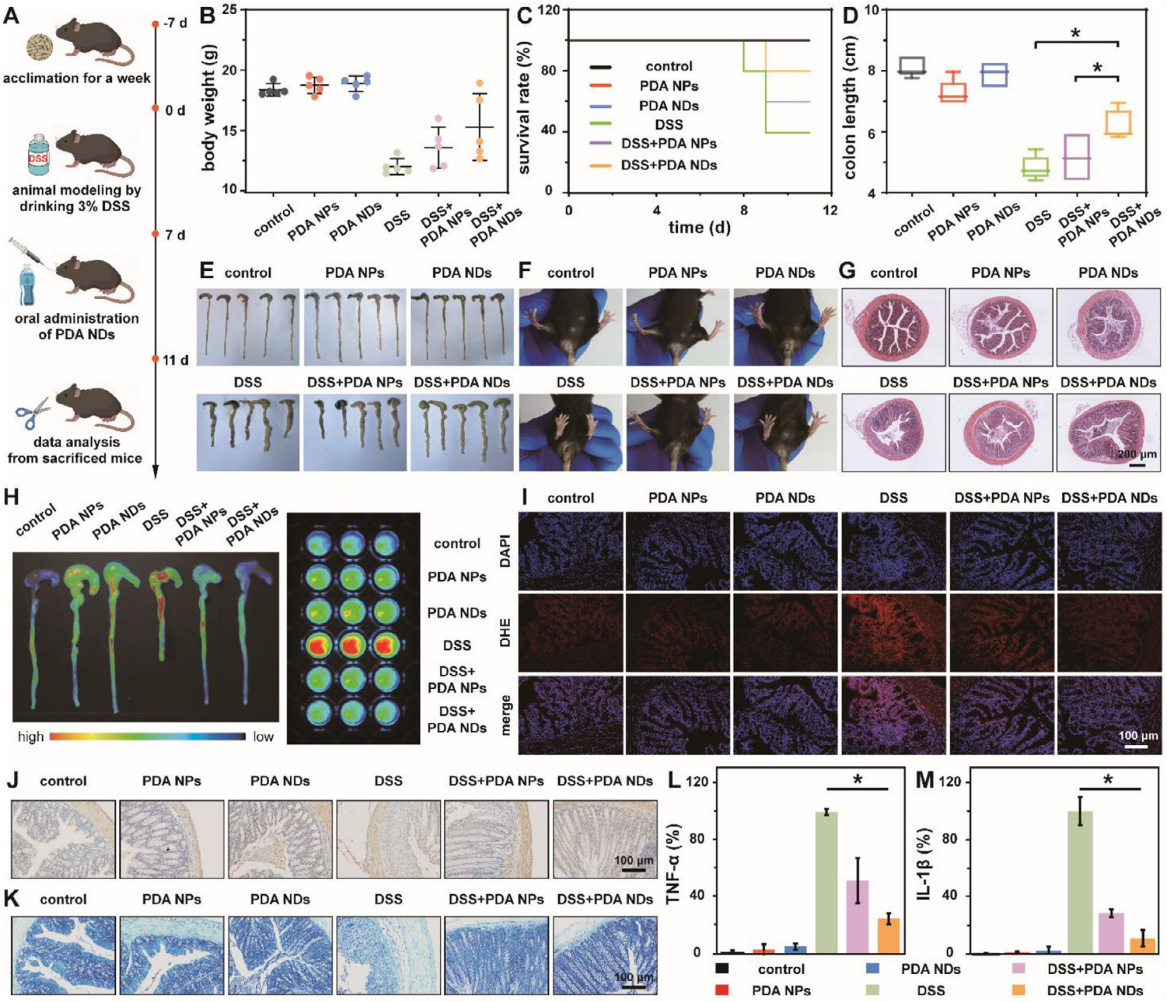
Schematic illustration of the establishment of DSS‐induced acute colitis and the corresponding treatment in mice (A). Changes in body weight (B) and time‐dependent survival rate (C) of mice after different treatments. Colon length (D) and the corresponding photos of colonic tissues (E) obtained from mice after different treatments. Photos of the anus in different groups (F). H&E staining images of the colonic tissue specimens from mice after different treatments (G). Fluorescence images and the corresponding quantitative analysis of ROS in colonic tissues from the mice after different treatments, indicated by DCFH‐DA (H). Fluorescence images of ROS in different colonic tissue specimens, indicated by DHE (I). Representative images of ZO‐1 (J) and TB (K) staining of the colonic tissue specimens from mice after different treatments. The levels of TNF‐α (L) and IL‐1β (M) in the colonic tissues from mice after different treatments. Data in (B) and (D) are presented as mean ± SD (*n* = 5). Data in (L) and (M) are presented as mean ± SD (*n* = 3). Statistical significance in (D) is calculated using unpaired *t*‐test. Statistical significance in (L) and (M) is calculated using one‐way ANOVA with multiple comparison tests. *p <0.05.

On the basis of the outstanding ROS scavenging activity, extremely low systemic toxicity, high stability in gastrointestinal tract, as well as admirable inflamed colon tissue targeting ability of PDA NDs, we further explored their in vivo therapeutic effects in murine models with IBDs. As the most common type of IBDs, ulcerative colitis (UC) was often accompanied with the excessive production of ROS.^[^ ^19^ ^]^ **Figure** **6**A illustrated the overall experimental design of PDA NDs in the treatment of acute UC. Mice were fed with DSS (3 wt.% in water) for a week to induce acute UC. The successful establishment of animal model was indicated by the obvious body weight loss and fecal occult blood for all DSS‐involved groups. At the end of experiment, the body weights of all groups were recorded and presented in Figure 6B. Compared with the healthy mice, DSS‐induced acute colitis mice showed significant weight loss. After the oral administration of PDA NDs or PDA NPs, the loss of mouse body weight could be relieved with different degrees. Significantly, the therapeutic efficacy of PDA NDs was much higher than that of PDA NPs. The survival percentages of mice receiving different treatments during the whole experimental period were shown in Figure 6C. As expected, PDA NDs held a better therapeutic effect and a lower mortality rate than PDA NPs while mice in the DSS group had the highest mortality rate of 60%. Colon length was considered as another critical factor to assess the therapeutic efficacy of PDA NDs. The colons of mice were collected immediately upon death, as well as measured and photographed at the end of the whole experiment. Figure 6D,E revealed that mouse colon lengths in the groups of control, PDA NPs, PDA NDs, DSS, DSS+PDA NPs, and DSS+PDA NDs were 8.16 ± 0.32, 7.30 ± 0.41, 7.92 ± 0.40, 4.90 ± 0.38, 5.24 ± 0.75, and 6.28 ± 0.47 cm, respectively. Among all the groups, the DSS‐treated mice held the shortest colon length. Meanwhile, PDA NDs provided mice the better therapeutic efficacy against DSS‐induced shortening of colon length than PDA NPs. Moreover, the photos of mouse anus, behavior, and bedding also represented that the mice in the DSS group seriously suffered from soft feces, diarrhea, and even bloody stools while PDA NDs could well alleviate the above symptoms in mice (Figure 6F; Figure S14A,B, Supporting Information). To better understand the therapeutic effect of PDA NDs, a scoring system for the status of DSS‐induced acute colitis mice was developed to quantify various damage indicators including weight loss, colon length, fecal viscosity, and hematochezia (Table S4, Supporting Information). As shown in Figure S14C,D (Supporting Information), it was easily found that PDA NDs played an essential role during the therapeutic process of UC, which could significantly alleviate the UC symptoms in mice and improve their life quality. The histopathological effect of PDA NDs in the treatment of DSS‐induced acute colitis was further evaluated using hematoxylin and eosin (H&E) staining (Figure 6G; Figure S14E, Supporting Information). For the DSS‐induced acute colitis mice, the intrinsic structure of colonic tissue disrupted and the colonic epithelium was severely damaged. However, improved histological appearance was easily detected in the DSS+PDA NDs group, suggesting that PDA NDs could preserve the integrity of colonic epithelium, reduce the infiltration of inflammatory cells, and alleviate colonic inflammation. Moreover, the DCFH‐DA perfusion combined with fluorescence imaging was utilized to evaluate the antioxidant effect of PDA NDs in the treatment of DSS‐induced acute colitis. Fluorescence images of colonic tissues from mice after different treatments revealed that the DSS group had the strongest fluorescence signal, indicating the high level of ROS in the damaged colons. Compared with the DSS group, the fluorescence signals in the DSS+PDA NPs group and DSS+PDA NDs group were significantly weakened, demonstrating the inhibition effect on the ROS generation (Figure 6H). Considering the possible autofluorescence of intestinal contents in the collected tissues from different groups, we further performed fluorescence imaging and quantitative analysis on the homogenized colonic tissues. Compared with PDA NPs, PDA NDs held better ROS scavenging activity and more excellent therapeutic efficacy against DSS‐induced acute colitis (Figure S15A, Supporting Information). Meanwhile, the dihydroethidium (DHE) staining was utilized to confirm the ROS scavenging performance of PDA NDs at the tissue level. As expected, the DSS group held the strongest fluorescence signal while the oral administration of PDA NDs could extremely reduce the ROS content in colonic tissues (Figure 6I; Figure S15B, Supporting Information). Besides that, the integrity of colonic epithelial barrier was further estimated by using immunohistochemical staining of intestinal mucins, such as zonula occludens‐1 (ZO‐1). Figure 6J revealed that the colonic epithelial barrier from mice in the DSS group completely disappeared, and those of mice in the other groups (except the groups of control, PDA NPs, and PDA NDs) exhibited different degrees of damage. However, the oral administration of PDA NDs could effectively preserve the colonic epithelial barrier integrity and alleviate the damage of colonic tissue in the mice with DSS‐induced acute colitis. In general, the activation of mast cells in IBDs could lead to the release of abundant pro‐inflammatory cytokines, which might cause sustained inflammation and immune activation in the intestine. As shown in Figure 6K, the images of colonic tissues stained with toluidine blue (TB) indicated that the mast cell number decreased after the oral administration of PDA NPs and PDA NDs when compared with the DSS group. To well understand the therapeutic mechanism of PDA NDs against DSS‐induced acute colitis, the levels of inflammation‐associated cytokines in colonic tissues were evaluated with the help of enzyme‐linked immunosorbent assay (ELISA). The oral administration of PDA NDs could highly reduce the levels of pro‐inflammatory cytokines, such as tumor necrosis factor‐α (TNF‐α) and interleukin‐1β (IL‐1β), indicating the potent anti‐inflammatory effect after PDA NDs‐assisted antioxidant therapy (Figure 6L,M). Meanwhile, the levels of colonic superoxide dismutase (SOD) and malondialdehyde (MDA) were quantified in the above groups. Compared to the control group, the SOD level of the DSS group decreased sharply, implying severe impairment of the antioxidant system in the colonic tissue in the DSS‐induced acute colitis mice (Figure S16, Supporting Information). After the oral administration of PDA NDs, the damaged antioxidant system could be relieved and reconstructed. Moreover, MDA could serve as an indicator of oxidative stress severity in IBDs due to the fact that the lipids oxidized by ROS usually resulted in a high concentration of MDA around the damaged colonic tissue. Our results revealed that the colonic tissues of the DSS group contained a large amount of MDA, which could be significantly decreased in the DSS+PDA NPs and DSS+PDA NDs groups. Compared with PDA NPs, our well‐developed PDA NDs could better alleviate the damage of colonic tissues in the mice with DSS‐induced acute colitis.

**Figure 7:**
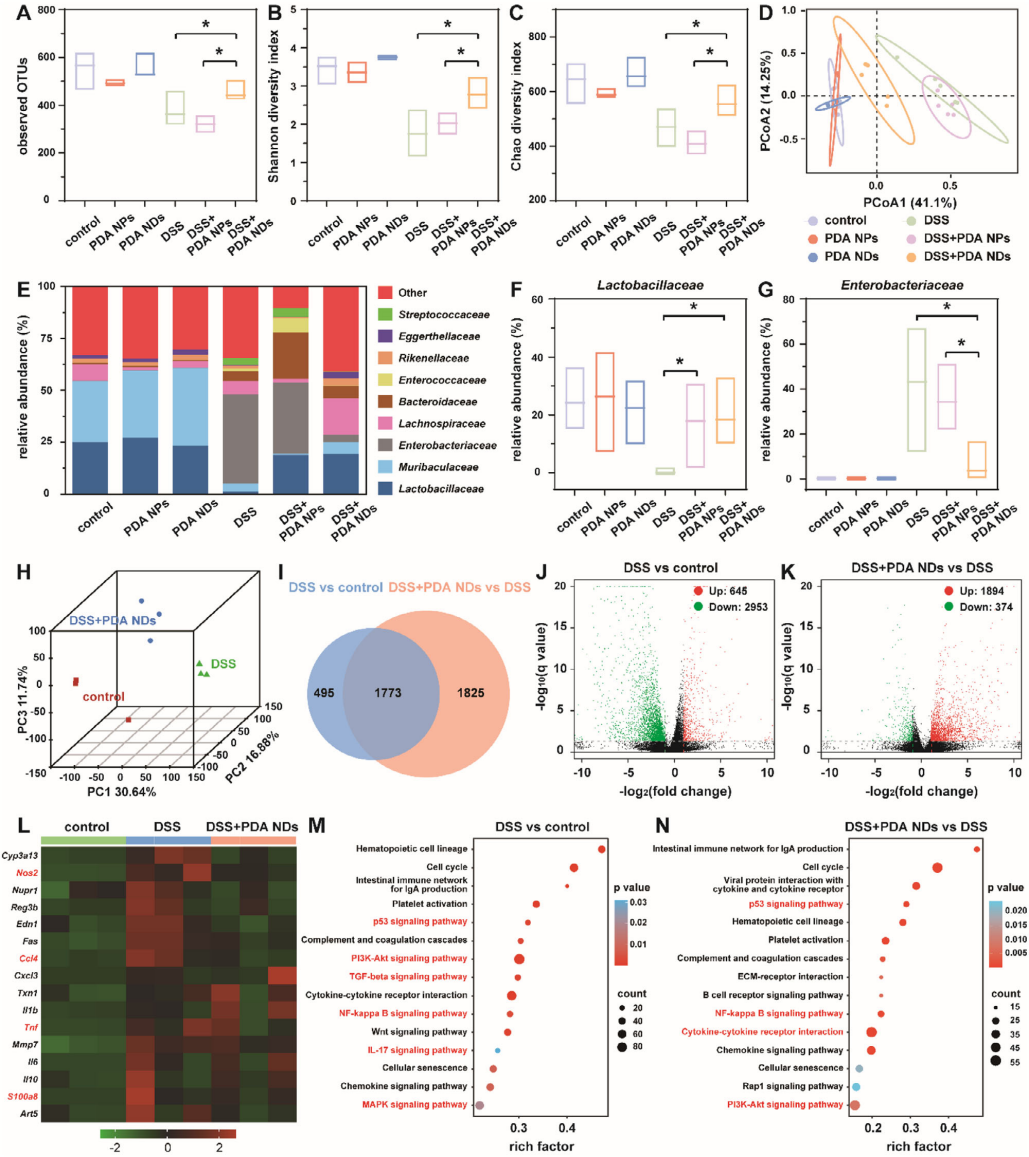
Estimation of microbial community observed operational taxonomic unit (OTU) richness (A). Alpha‐diversity was displayed using Shannon diversity index (B) and Chao diversity index (C). PCoA indicating the β‐diversity of gut microbiome (D). Relative abundance of the gut microbiome at family‐level taxonomy articulated as a proportion of all sequences (E). Relative abundance of selected taxa including *Lactobacillaceae* (F) and *Enterobacteriaceae* (G). PCA of the genes in healthy and IBD mice with and without the treatment of PDA NDs (H). Venn diagram indicating the intersection target genes of mice after different treatments (I). Volcano plot showing the differentially expressed genes in the comparison of DSS with the healthy group (J) and the DSS group treated with PDA NDs versus the DSS group (K). Heatmap of RNA‐seq data showing differently expressed genes among different groups after cluster analysis (L). KEGG pathway enrichment analysis of the differentially expressed genes in the comparison of DSS with the healthy group (M) and in the DSS group treated with PDA NDs versus the DSS group (N). Data in (A), (B), (C), (F), and (G) are presented as mean ± SD (*n* = 5). Statistical significance is calculated using unpaired *t*‐test. *p <0.05.

As well known, the dysbiosis of gut microbiome including reduced diversity and altered microbiota composition was usually found during the occurrence and development of IBDs. Given the favorable therapeutic effect of PDA NDs, we further explored their positive role in the modulation of gut microbiome in vivo by using 16S rRNA sequencing of fecal samples. As evidenced in observed operational taxonomic units (OTUs) and α‐diversity indices including Shannon and Chao, the bacterial richness and diversity were significantly reduced in the IBD mice (**Figure** **7**A–C). After the treatment of PDA NPs, the above‐mentioned undesirable phenomenon was not efficiently alleviated. However, the intervention of PDA NDs could well improve the bacterial richness and α‐diversity. Meanwhile, the differences in microbiome composition among groups were investigated using β‐diversity analysis. As shown in Figure 7D, the results of principal coordinate analysis (PCoA) indicated that the DSS+PDA NDs group held a distinct intestinal microbiota profile compared to the groups of DSS and DSS+PDA NPs. Moreover, the DSS+PDA NDs group and the groups of control, PDA NDs, and PDA NPs clustered closely, suggesting that PDA NDs could restore gut microbiome to a healthy state. Subsequently, the effects of PDA NDs on gut microbiota composition were well investigated. Detailed information at the family level revealed the bacterial composition of treated IBD mice (Figure 7E). Obviously, the treatment of PDA NDs could significantly increase the relative abundance of *Lactobacillaceae*, which was negatively associated with the inflammatory state of IBDs (Figure 7F). As a positive indicator for the inflammatory state of IBDs, the relative abundance of *Enterobacteriaceae* was remarkably decreased after the treatment of PDA NDs (Figure 7G). A taxonomic cladogram generated from Linear discriminant analysis Effect Size (LEfSe) at the genus level re‐confirmed the above observations, showing a reduction in pathogenic bacteria and an enrichment of beneficial bacterial species after the treatment of PDA NDs (Figure S17, Supporting Information). Compared to PDA NPs, the treatment of PDA NDs could significantly decrease the relative abundance of *Escherichia‐Shigella*, which was well known for producing extracellular toxins and inducing intestinal inflammation (Figure S18, Supporting Information). Besides, PDA NDs could also specifically contribute to a significant increase in the abundance of *Lactobacillus*. Therefore, PDA NDs could well modulate the gut microbiota composition by enhancing the bacterial diversity and restoring the relative abundance of beneficial bacteria in IBD mice.

To further evaluate the therapeutic mechanism of PDA NDs on IBD mice, colon tissues were collected after the PDA NDs treatment for transcriptomic analysis. Figure 7H revealed that the resulting principal component analysis (PCA) diagram well clustered the samples, segregating the DSS group from both the control and DSS+PDA NDs group. Meanwhile, the Venn diagram identified the distinct transcriptome profiles of the control, DSS, and DSS+PDA NDs groups (Figure 7I). Moreover, differentially expressed genes (DEGs) exhibited a clear clustering and volcano plots highlighted significant differences between the two groups. Compared to the healthy mice, there were 645 up‐regulated genes and 2953 down‐regulated genes in the IBD mice (Figure 7J). Following the treatment of PDA NDs, 1894 genes were up‐regulated and 374 genes were downregulated (Figure 7K). Subsequently, the gene expression differences among the control, DSS and DSS+PDA NDs groups were represented using a normalized heatmap (Figure S19, Supporting Information). Compared to the healthy mice, several critical pro‐inflammatory genes including CCL4, TNF, NOS2, and S100A8 were evidently up‐regulated in the IBD mice, which were decreased after the treatment of PDA NDs (Figure 7L). Similarly, it could be easily found that the genes related to ROS in IBD mice were down‐regulated after the PDA NDs treatment. In addition, the Gene Ontology (GO) analysis of DEGs in the colonic tissues from mice after different treatments was performed to confirm their functional relevance (Figure S20, Supporting Information). Compared to the healthy mice, DEGs achieved from the IBD mice were mainly enriched in the biological processes related to the ROS generation and inflammation. After the treatment of PDA NDs, the regulation of response to ROS and inflammatory response became prominent, which was highly consistent with the other results of transcriptomic analysis. Besides, the Kyoto Encyclopedia of Gene and Genomes (KEGG) enrichment analysis showed that the p53 signaling pathway, cytokine–cytokine receptor interaction, PI3K‐Akt signaling pathway, and NF‐κB signaling pathway were overexpressed in the IBD mice in comparison with the healthy mice or PDA NDs‐treated IBD mice, suggesting their direct association with the therapeutic mechanisms of PDA NDs (Figure 7M,N). Therefore, the above evidence indicated that the treatment of PDA NDs could effectively eliminate the intestinal oxidative stress, inhibit inflammation‐related signaling pathways, reduce the expression of pro‐inflammatory cytokines, as well as restore the intestinal barrier function.

Given their favorable therapeutic effects toward DSS‐induced acute colitis in mice, we further evaluated the performance of PDA NDs in comparison to clinical medications. During the experiment, 5‐aminosalicylic acid (5‐ASA) was selected as the typical positive drug, which was widely utilized for treating colitis in daily clinic. Figure S21A (Supporting Information) illustrated the experimental design of PDA NDs in the treatment of acute colitis in the presence of 5‐ASA. Compared with the healthy mice, DSS‐induced acute colitis mice showed significant weight loss. After the oral administration of PDA NDs, PDA NPs, or 5‐ASA, the loss of body weight was relieved with different degrees (Figure S21B, Supporting Information). Notably, the therapeutic effect of PDA NDs was highest among all the treatment groups. Meanwhile, PDA NDs provided mice the best therapeutic performance against DSS‐induced shortening of colon length among all the treatment groups (Figure S21C, Supporting Information). According to the result of H&E staining, improved histological appearance was easily found in all the treatment groups and the group of DSS+PDA NDs held the best therapeutic effect (Figure S21D, Supporting Information). Moreover, DHE staining was utilized to confirm the ROS scavenging performance of PDA NPs, 5‐ASA, and PDA NDs at the tissue level. As shown in Figure S21E (Supporting Information), the oral administration of PDA NDs achieved a higher ROS clearance efficiency than both PDA NPs and 5‐ASA. Significantly, the daily dosage of PDA NDs was 20 mg kg^−1^, which was much lower than that of 5‐ASA (50 mg kg^−1^). All these evidence suggested that PDA NDs could better eliminate ROS in intestinal cells and alleviate intestinal damage as compared with 5‐ASA.

### 2.7 Treatment of DSS‐Induced Chronic Colitis

**Figure 8:**
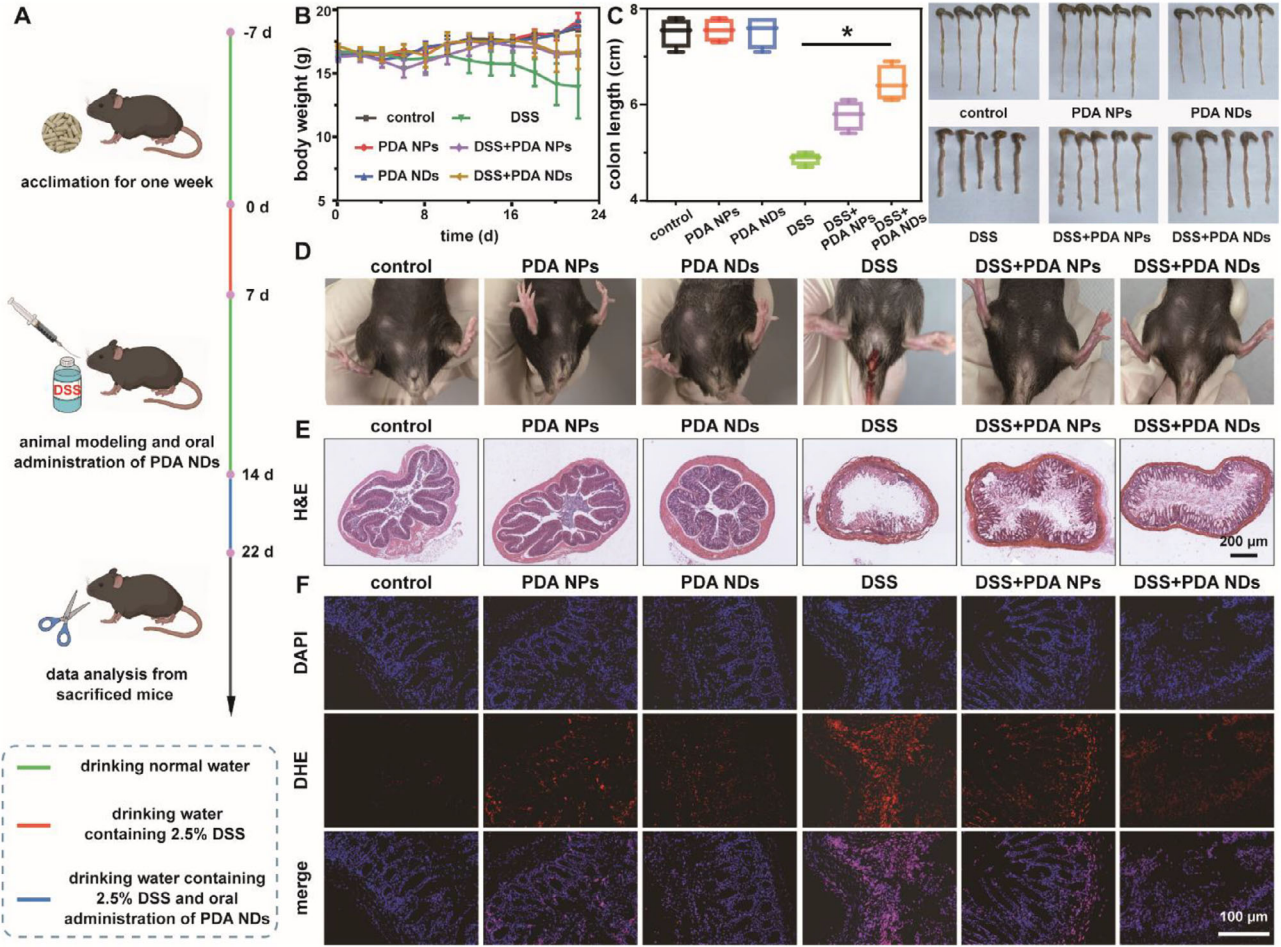
Schematic illustration of the establishment of DSS‐induced chronic colitis and the corresponding treatment in mice (A). Changes in body weight of mice after different treatments (B). Colon length and the corresponding photos of colonic tissues obtained from mice after different treatments (C). Photos of the anus in different groups (D). H&E staining images of the colonic tissue specimens from mice after different treatments (E). Fluorescence images of ROS in different colonic tissue specimens, indicated by DHE (F). Data in (B) and (C) are presented as mean ± SD (*n* = 5). Statistical significance is calculated using one‐way ANOVA with multiple comparison tests. *p <0.05.

Due to the excellent therapeutic outcomes of PDA NDs on the mice with DSS‐induced acute colitis, we further explored their performance on the mice with DSS‐induced chronic colitis. **Figure** **8**A illustrated the overall experimental design of PDA NDs in the treatment of chronic colitis. Compared to the normal mice in the control group, the body weight of mice with chronic colitis was highly reduced, which was also utilized to indicate the successful onset of animal model (Figure 8B). Throughout the whole experimental period, mice in the groups of DSS+PDA NPs and DSS+PDA NDs maintained higher body weight than those with DSS‐induced chronic colitis, showing effective weight recovery. As shown in Figure 8C, colon lengths in the groups of control, PDA NPs, PDA NDs, DSS, DSS+PDA NPs, and DSS+PDA NDs were 7.5±0.29, 7.55±0.24, 7.53±0.34, 4.88±0.13, 5.88±0.30, and 6.35±0.37 cm, respectively. It was worthy of note that each treatment group exhibited more or less therapeutic effects on the colon length reduction caused by chronic colitis while the DSS+PDA NDs group could return to a level more close to that of the control group. Because of the characteristics of chronic colitis, no mouse died at the endpoint of the experiment among all groups (Figure S22A, Supporting Information). Evidenced by the photos of mouse anus, fece, and behavior, mice with DSS‐induced chronic colitis seriously suffered from the soft feces and bloody stools while PDA NDs could well alleviate the above symptoms in mice (Figure 8D; Figure S22B,C, Supporting Information). To precisely determine the degree of colonic injury, a scoring system for the status of DSS‐induced chronic colitis mice was utilized to quantify various damage indicators including weight loss, colon length, stool viscosity, and hematochezia (Table S5, Supporting Information). According to our scoring system, it could be easily found that PDA NDs played an essential role during the therapeutic process of DSS‐induced chronic colitis, which could well relieve the symptoms of chronic colitis and improve the quality of life in mice (Figure S22D,E, Supporting Information). Histological analysis provided additional insight into the severity of colonic epithelial damage (Figure 8E; Figure S22F, Supporting Information). Significantly, severe colonic damage with aberrant crypt structures was obvious in the mice with DSS‐induced chronic colitis. Different degrees of recovery from colon epithelial damage were observed across the groups of DSS+PDA NDs and DSS+PDA NPs while the PDA NDs group held a colonic tissue structure similar to that of the control group. Furthermore, the DHE staining was utilized to confirm the ROS scavenging performance of PDA NDs at the tissue level. Figure 8F and Figure S22G (Supporting Information) revealed that the DSS group exhibited the strongest red fluorescence signal, indicating the highest level of ROS in the colonic tissue. In contrast, the red fluorescence signals in the groups of DSS+PDA NPs and DSS+PDA NDs were highly weakened compared with that in the DSS group, suggesting the effective ROS removal and excellent therapeutic outcome.

After understanding their favorable therapeutic effects toward DSS‐induced chronic colitis in mice, we further evaluated the effect of PDA NDs in comparison to 5‐ASA. Detailed experimental design of PDA NDs in the treatment of chronic colitis was illustrated in Figure S23A (Supporting Information). As expected, the loss of body weight could be relieved with different degrees in all the treatment groups, whereas PDA NDs held the best therapeutic effect (Figure S23B, Supporting Information). Meanwhile, PDA NDs provided mice the best therapeutic performance against DSS‐induced shortening of colon length among all the treatment groups (Figure S23C, Supporting Information). According to the results of H&E staining, PDA NDs held better therapeutic effect than 5‐ASA in improving histological appearance (Figure S23D, Supporting Information). More importantly, the oral administration of PDA NDs achieved a higher ROS clearance efficiency than both PDA NPs and 5‐ASA, which was evidenced by the results of DHE staining (Figure S23E, Supporting Information). These exciting results thus implied that PDA NDs exhibited better therapeutic performance than 5‐ASA in the treatment of DSS‐induced chronic colitis.

### 2.8 Treatment of TNBS‐Induced Crohn's Disease

**Figure 9:**
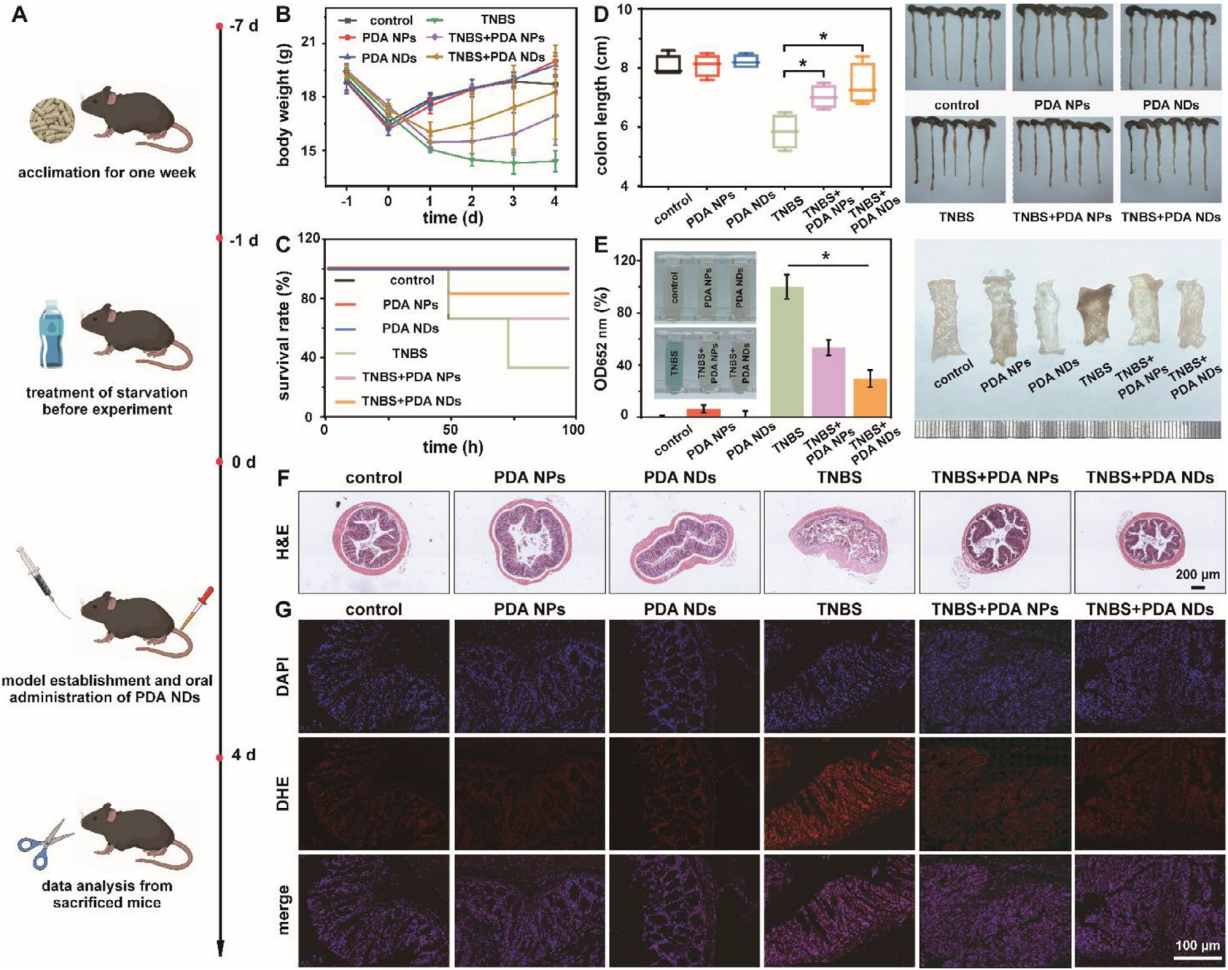
Schematic illustration of the establishment of TNBS‐induced colitis and the corresponding treatment in mice (A). Change in body weight (B) and time‐dependent survival rate (C) of mice after different treatments. Colon length and the corresponding photos of colonic tissues from mice after different treatments (D). Analysis of colonic occult blood and the corresponding photos of colonic tissues from mice after different treatments (E). H&E staining images of the colonic tissue specimens from mice after different treatments (F). Fluorescence images of ROS in different colonic tissue specimens, indicated by DHE (G). Data in (B), (D), and (E) are presented as mean ± SD (*n* = 6). Statistical significance in (D) is calculated using unpaired *t*‐test. Statistical significance in (E) is calculated using one‐way ANOVA with multiple comparison tests. *p <0.05.

To explore the breadth of therapeutic usages of PDA NDs, we further evaluated their efficacy as a therapy for Crohn's disease (CD).^[^ ^20^ ^]^ Typically, mice were rectally administrated with 2,4,6‐trinitro benzene sulfonic acid (TNBS) to establish the CD model. **Figure** **9**A summarized the overall experimental design of PDA NDs in the treatment of TNBS‐induced CD. Differences of body weight, mortality rate, and colon length indicated the excellent therapeutic efficacy of PDA NDs. Compared with the mice in the groups of TNBS and TNBS+PDA NPs, mice in the TNBS+PDA NDs group had less weight loss, implying that PDA NDs could well ameliorate the progression of TNBS‐induced CD (Figure 9B). Moreover, mice in the TNBS+PDA NDs group had the lowest mortality rate among all the TNBS‐involved groups (Figure 9C). The colon lengths in the groups of control, PDA NPs, PDA NDs, TNBS, TNBS+PDA NPs, and TNBS+PDA NDs were 8.05±0.38, 8.10±0.39, 8.23±0.26, 5.85±0.60, 7.03±0.40 and 7.43±0.71 cm, respectively (Figure 9D). Accordingly, PDA NDs could extremely attenuate the shortening of colon length in TNBS‐treated mice. Figure 9E revealed that mouse feces in the TNBS group exhibited strong peroxidase‐like activity, demonstrating the presence of fecal occult blood in CD mice. After the treatment of PDA NDs, the degree of fecal occult blood in CD mice was highly reduced, corresponding to the photos of bedding (Figure S24A, Supporting Information). These results implied that PDA NDs were more effective at inhibiting the colonic tissue bleeding in CD mice, which was highly consistent with our observations on the colonic tissues of healthy and CD mice. A scoring system for the status of TNBS‐induced CD mice was then utilized to comprehensively assess the therapeutic performance of PDA NDs (Table S6, Supporting Information). The resulting radar chart and disease activity index indicated that PDA NDs could efficiently protect the mice from TNBS‐induced colonic injury (Figure S24B,C, Supporting Information). According to the histological analysis, CD mice showed severe mucosal destruction, massive inflammatory cell infiltration, and colonic epithelium damage while the PDA NDs treatment could well repair epithelial cells and reconstruct crypt structure with great therapeutic outcome (Figure 9F; Figure S24D, Supporting Information). The degree of colonic damage in different groups was further assessed using Terminal Deoxynucleotidyl Transferase‐mediated dUTP Nick‐End Labeling (TUNEL) immunohistochemical staining. Evidence by Figure S24E (Supporting Information), the apoptotic level of colonic epithelial cells in the TNBS+PDA NDs group was highly reduced, similar to that observed in healthy mice. Last but not least, DHE staining and relative quantitative analysis demonstrated that PDA NDs exhibited high‐performance ROS scavenging activity and admirable therapeutic efficacy in the treatment of TNBS‐induced CD (Figure 9G; Figure S24F, Supporting Information). Together, well‐prepared PDA NDs could efficiently reduce the oxidative stress and apoptosis in colonic tissue, well alleviating TNBS‐induced CD in mice.

## 3 Conclusion

In summary, this study demonstrated the great potential of ultrasmall polydopamine nanodots as a novel oral formulation for different degrees of IBDs by scavenging excessive ROS around intestinal lesions and regulating proinflammatory microenvironment. As expected, the designed PDA NDs not only held excellent broad‐spectrum antioxidant activity but also exhibited high stability in the harsh gastrointestinal environment. Moreover, the negatively charged surface of PDA NDs endowed them with an outstanding targeting capability toward the positively charged intestinal inflamed lesions via electrostatic interactions. Benefiting from these, oral administration of PDA NDs showed admirable therapeutic performance in acute and chronic ulcerative colitis, as well as Crohn's disease models. The therapeutic mechanism of PDA NDs mainly involved the elimination of oxidative stress, the reduction of ROS‐mediated proinflammatory response, the amelioration of intestinal barrier function, and the restoration of normal gut microbiota. Last but not the least, long‐term toxicity exploration of PDA NDs after oral administration indicated their high biocompatibility and safety. Together, our current findings proposed a new ROS‐ and inflammation‐scavenging approach to treat IBD, while providing further inspiration for the design of novel oral formulations with great clinical translation prospects.

## Conflict of Interest

The authors declare no conflict of interest.

## Supporting information



Supporting Information

## Data Availability

The data that support the findings of this study are available from the corresponding author upon reasonable request.
