# Supplementary material for: Polydopamine Nanodots Ameliorate Inflammatory Bowel Disease by Restoring Redox Homeostasis and Intestinal Microenvironment
Source: Adv Sci (Weinh). 2025 Aug 23;12(47):e08674. doi: 10.1002/advs.202508674 (PMC12713062; doi:10.1002/advs.202508674)
Supplement: Supplementary file 1 — Supporting Information [file ADVS-12-e08674-s001.pdf]

Supporting Information for

Polydopamine Nanodots Ameliorate Inflammatory Bowel Disease by Restoring Redox Homeostasis and Intestinal Microenvironment

Zhen Ding, Xingfu Bao, Ying Zhao, Lin Bai, Jinming Zhang, Chengjing Xu, Tianyan Chen, Shuang Dai, Yufu Liu, Min Hu, Meng Qin*, and Zhen Liu**

1. Experimental section

Chemicals. Dopamine hydrochloride, methionine, riboflavin, (3-aminopropyl) triethoxysilane (APTES), methylene blue (MB), nitrotetrazolium blue chloride (NBT), thiobarbituric acid (TBA), 2,2'-azinobis(3-ethylbenzothiazoline-6-sulfonic acid ammonium salt) (ABTS), 2,2-diphenyl-1-(2,4,6-trinitrophenyl) hydrazyl (DPPH), 2-phenyl-4,4,5,5-tetramethylimidazoline-3-oxide-1-oxyl radical (PTIO), 5,5-dimethyl-1-pyrroline N-oxide (DMPO), 2',7'-dichlorodihydrofluorescein diacetate (DCFH-DA), dihydroethidium (DHE), dextran sulfate sodium salt (DSS), and tris(hydroxymethyl)aminomethane (Tris) were purchased from Aladdin Reagent (Shanghai, China). Fluorescein isothiocyanate (FITC)-labeled phalloidin, 2,4,6-trinitro-benzenesulfonic acid (TNBS), 3-(4,5-dimethyl-2-thiazolyl)-2,5-diphenyl-2H-tetrazolium bromide (MTT), 4',6-diamidino-2-phenylindole (DAPI), and 5-aminosalicylic acid (5-ASA) were achieved from Sigma-Aldrich (Shanghai, China). Dulbecco's Modified Eagle Medium (DMEM), RPMI-1640 medium, and fetal bovine serum (FBS) were acquired from Sangon (Shanghai, China). Lactate Dehydrogenase (LDH) Assay Kit, Calcein AM/Propidium Iodide (PI) Cell Viability Assay Kit, Reactive Oxygen Species (ROS) Assay Kit, β-galactosidase Assay Kit, DNA Damage Assay Kit by γ-H2AX immunofluorescence, and Annexin V-FITC/PI Apoptosis Detection Kit were purchased from Beyotime (Shanghai, China). Malondialdehyde (MDA) Assay Kit and Superoxide Dismutase (SOD) Assay Kit were obtained from Nanjing Jiancheng Bioengineering Institute (Nanjing, China). Other chemicals were obtained from Beijing Chemicals (Beijing, China). All chemicals were of analytical grade and utilized without any purification. Water throughout all experiments was prepared using a Milli-Q water system.

Cell culture. Caco-2 cells acquired from Shanghai FUHENG Biotechnology Co., Ltd (Shanghai, China) were cultured in RPMI-1640 medium containing penicillin (100 U/mL), streptomycin (100 U/mL), and 10% FBS in a humidified incubator at 37°C and 5% CO_2. RAW 264.7 cells purchased from Chinese Academy of Medical Sciences (Beijing, China) were cultured in DMEM containing 10% FBS in a humidified incubator at 37°C with 5% CO_2. For the following studies, cells were

harvested by trypsinization and re-suspended in fresh medium containing FBS before plating.

Animals. C57BL/6J mice and BALB/cA-nu mice were purchased from Beijing Huafukang Bioscience Co., Ltd (Beijing, China). All animal care and experimental procedures were under institutional policies for animal health and wellbeing, and were approved by the Institutional Animal Care and Use Committee of Jilin University (SY202103014).

Instrumentations. Transmission electron microscopy (TEM) images were captured on a FEI TECNAI G2 F20 S-TWIN transmission electron microscope. Scanning electron microscopy (SEM) image was achieved on an Apreo 2C scanning electron microscope. Atomic force microscope (AFM) image was recorded in the tapping mode with a Dimension ICON scanning probe microscope. UV-vis spectra were recorded on a Lambda 950 UV-vis spectrophotometer. For the information of chemical structure and functional groups, samples were scanned on a Bruker Vertex 70 FT-IR spectrometer. X-ray photoelectron spectroscopy (XPS) spectra were measured on an ESCALAB 250Xi photoelectron spectrometer. Zeta potentials were measured on a Malvern Nano ZS-90. Raman spectra were recorded on a LabRAM HR Evolution Raman spectrometer. ^{13}C NMR spectra were captured on a Bruker Advance III 400 MHz spectrometer. Electron spin resonance (ESR) spectra of various products were recorded on a Bruker A200 while ESR spectra of $\cdot OH$ and $\cdot O_2^-$ scavenging were recorded on a Bruker EMX-500 10/12 spectrometer at room temperature. Cellular images were acquired on a laser scanning confocal microscopy (Leica TCS SP5 II). Intracellular ROS scavenging assay and anti-apoptotic effect of nanoparticles were performed on a flow cytometer (BD FACSCalibur).

Synthesis of PDA NDs. Polydopamine nanodots (PDA NDs) were synthesized using a mild hydrothermal approach. Typically, dopamine hydrochloride (2 g) was added into deionized water (40 mL) under stirring, Subsequently, above mixture was transferred into a Teflon-lined autoclave and heated at 180°C for 12 h. The resulting solution was dialyzed against deionized water. PDA NDs were collected after removing the large particles by centrifugation and re-dispersed in deionized water for further use.

Synthesis of Cy5.5-modified PDA NDs. Briefly, PDA NDs (4 mg) and Cy5.5-PEG-NH_2 with a molecular weight of 2000 (2 mg) were mixed in Tris buffer (pH 8.5, 10 mM). Above reaction was allowed to proceed overnight in the dark under stirring. Final products were dialyzed against deionized water and freeze-dried for further use.

Synthesis of PDA NPs. Polydopamine nanoparticles (PDA NPs) were synthesized according to a previous study with some modifications. Typically, concentrated ammonia aqueous solution (2.5 mL, NH_4OH, 28-30%) was mixed with ethanol (40 mL) and deionized water (90 mL) under stirring. 0.5 h later, solution containing dopamine hydrochloride (0.05 g/mL, 10 mL) was added into the above mixture. 24 h later, PDA NPs were obtained after centrifugation and washed with deionized water for further use.

·OH scavenging assay. Methylene blue (MB) was utilized as the typical colorimetric reagent for the detection of ·OH. The protective effect of PDA NDs on the decolorization of MB in the presence of ·OH was taken as an indicator of their antioxidant activity. In common, ·OH could be generated by the decomposition of H_2O_2. The absorbance values of mixture with a volume of 4 mL containing MB (50 μg/mL), $FeSO_4$ (1 mM), and H_2O_2 (2 mM) in the presence of PDA NDs with different concentrations were recorded on a UV-vis spectroscopy. Without the addition of PDA NDs, MB solution could be totally bleached by the generated ·OH based on our current design. Moreover, ·OH scavenging activity was measured using an ESR spectroscopy with the help of DMPO as a capture reagent. Typically, a sample (200 μL) containing H_2O_2 (2.5 mM), $FeSO_4$ (20 μM), PDA NDs (0.2 mg/mL), and DMPO (10 μL) was prepared in neutral PBS buffer, and ESR signals were monitored on a Bruker EMX-500 10/12 spectrometer.

$\cdot O_2^-$ scavenging assay. $\cdot O_2^-$ scavenging activity of PDA NDs was achieved by quantifying the inhibition ratio of photo-reduction of NBT. Mixtures containing riboflavin (20 μM), NBT (75 μM), methionine (13 mM), and different concentrations of PDA NDs were prepared in neutral PBS buffer (25 mM, 4 mL). Subsequently, the above mixtures were placed under an incandescent lamp light. After the illumination, absorbance values of the above mixtures at 560 nm were recorded, and the

corresponding photos were collected. The solution containing riboflavin, NBT, and methionine without illumination was defined as the negative control while the solution containing riboflavin, NBT, and methionine after illumination was defined as the positive control. Furthermore, $\cdot O_2^-$ scavenging activity of PDA NDs was reconfirmed using an EPR spectroscopy with the help of DMPO. For the generation of $\cdot O_2^-$, riboflavin (200 μM) was added into methanol under illumination. For $\cdot O_2^-$ scavenging, a sample (200 μL) containing riboflavin (200 μM), PDA NDs (0.2 mg/mL), and DMPO (10 μL) was prepared in methanol. After the illumination, PDA NDs and DMPO were added into the above mixture. 5 min later, ESR signals were monitored on a Bruker EMX-500 10/12 spectrometer.

DPPH· scavenging assay. Briefly, DPPH· solution (0.5 mg/mL, 0.4 mL) was mixed with ethanol solution (3.6 mL) containing different concentrations of PDA NDs for 2 h in the dark at first. Subsequently, the absorbance values at 519 nm were recorded on a UV-Vis spectroscopy, and the eliminated DPPH· was calculated at the same time.

$ABTS^{+}$· scavenging assay. Typically, an equal volume of ABTS diammonium salt (7.4 mM) and $K_2S_2O_8$ solution (2.6 mM) were mixed in the dark overnight. For the final $ABTS^{+}$· working solution, above mixture was diluted 20 times with deionized water until its absorbance value at 734 nm was lower than 1. Then, $ABTS^{+}$· working solution (0.4 mL) was mixed with different concentrations of PDA NDs, PBS (pH 7.4, 250 mM, 0.4 mL) was added, and the total volume was made up to 4 mL with deionized water. 2 h later, UV-vis spectroscopy was utilized to monitor the absorption spectrum with a range of 300-800 nm, and the corresponding absorbance values at 734 nm were recorded at the same time.

PTIO· scavenging assay. PTIO· solution (0.5 mg/mL, 0.4 mL) was mixed with PBS (pH 7.4, 250 mM, 0.4 mL) containing different concentrations of PDA NDs. Total volume of the above mixture was made up to 4 mL with the addition of deionized water. After stirring, the above mixture was kept in the dark overnight. UV-vis spectroscopy was utilized to scan the absorption spectrum with a range of 300-800 nm, and the corresponding absorbance values at 557 nm were recorded for the calculation of scavenging rates of PTIO·.

Stability of PDA NDs in simulated gastric fluid and simulated intestinal fluid. For the simulation of food digestion process in gastrointestinal tract, simulated gastric fluid (SGF) and simulated intestinal fluid (SIF) were prepared at first. For SGF, pepsin (3.2 g) and NaCl (2 g) were mixed with concentrated HCl, the pH value was adjusted to 1.2, as well as the final volume of above mixture was made up to 1 L with deionized water. For SIF, KH_2PO_4 (6.8 g) and trypsin (10 g) were mixed with deionized water (500 mL), the pH value was adjusted to 6.8 with the addition of NaOH, as well as the final volume of above mixture was made up to 1 L with deionized water. To explore their stability in different simulated fluids, PDA NDs were added into the above SGF or SIF, respectively. After the incubation at 37°C for 4 d, residual PDA NDs were collected, washed with deionized water, and allowed to proceed TEM analysis. In addition, DPPH· scavenging assay was utilized to determine the stability and the corresponding antioxidant activity of PDA NDs after various treatments. Last but not least, we explored the time-dependent changes of zeta potentials of PDA NDs after the treatments of SGF or SIF, respectively.

Charge-dependent target capability of PDA NDs in vitro. Generally, the charges of colon epithelium could change from negative to positive in the presence of colitis. In this study, slides with different charges were prepared to investigate the targeting capabilities of PDA NDs in vitro. Briefly, commercially available slides after ultrasonic cleaning were immersed into APTES solution (0.5%) for 2 h to create positively charged slides. Guided by the NHS/EDC-assisted coupling reaction, the above positively charged slides reacted with glutaric anhydride in Tris buffer to form negatively charged slides. Finally, three kinds of slides with different charges were co-incubated with Cy5.5-modified PDA NDs in the dark at 37°C for 3 h. Deionized water was utilized to remove the physically adsorbed PDA NDs, and fluorescent images were captured on an in vivo imaging system.

Cytotoxicity and LDH leaking assay. Caco-2 cells were seeded at a density of 5×10^3 cells/well in 96-well plates at first. After the cellular attachment, PDA NDs with different concentrations were added into the above wells and cultured for another 24 h. At the end of the incubation time, medium containing PDA NDs was removed, and cells were treated with MTT for another 4 h, which was

followed by the addition of DMSO to dissolve the formazan crystals. A microplate reader was utilized to measure the absorbance values of above wells at 490 nm. Meanwhile, LDH leakages from cells treated with different concentrations of PDA NDs were measured using a LDH Assay Kit.

Live/dead staining. Calcein AM/PI Cell Viability Assay Kit was utilized to detect the visible cytotoxicity of PDA NDs. Caco-2 cells were seeded in a 6-well plate with a density of 5×10^5 cells/well at first. After the cellular attachment, PDA NDs with different concentration were added into the above wells and cultured for another 24 h. At the end of the incubation time, medium containing PDA NDs was replaced with Calcein AM/PI working solution, and cells were allowed to be incubated in the dark for another 0.5 h. After rinsing with serum-free medium, fluorescence images were captured on a confocal microscope.

Hemolysis assay and coagulation assay. Fresh blood stabilized with heparin was collected and washed with 0.9% NaCl until the supernatant became clear. Red blood cells (RBCs) were diluted to 1/3 of their volume with 0.9% NaCl. Diluted RBCs (0.1 mL) were mixed with 0.9% NaCl (0.9 mL) as the negative group, water (0.9 mL) as the positive group, and 0.9% NaCl containing different concentrations of PDA NDs (0.9 mL) as the experimental samples. All the samples were vortexed and incubated at 37°C for 2 h. After centrifugation, the absorbance values of supernatants at 541 nm were measured. Hemolysis rate = (sample absorbance-negative absorbance)/(positive absorbance-negative absorbance). For the coagulation assays, plasma was mixed with 0.9% NaCl containing different concentrations of PDA NDs at first. Subsequently, a fully automatic blood coagulation analyzer was utilized to determine the values of activated partial thromboplastin time (APTT), prothrombin time (PT), thrombin time (TT), and fibrinogen (FIB).

Long-term toxicity of PDA NPs and PDA NDs. C57BL/6J mice after adaptive feeding were randomly divided into 3 groups, which were defined as control, PDA NPs and PDA NDs. PDA NPs and PDA NDs dispersed in 0.9% NaCl were orally administered to each mouse in the groups of PDA NPs and PDA NDs with a dosage of 100 mg/kg. Meanwhile, mice in the control group were orally administered with the same amount of 0.9% NaCl. Body weights and behavior of mice in the above

3 groups were recorded for 14 d. At the end of the whole experiment, mice were sacrificed, and urine, blood, as well as organs were individually collected for the following urinalysis, hematological analysis, biochemical assay, and histopathological analysis.

Bio-distribution of PDA NDs in vivo and ex vivo. BALB/cA-nu mice with inflammatory bowel disease (IBD) were achieved after oral administration of DSS. In detail, DSS (3%) was orally administrated to BALB/cA-nu mice for 5 d toward the development of IBD nude mice. In our current design, healthy mice were employed as the group of control. Cy5.5-modified PDA NDs (200 μg/mL, 1 mL) were orally administered to healthy and IBD mice, respectively. For the in vivo imaging, mice were imaged under an in vivo imaging system after oral administration of Cy5.5-modified PDA NDs at different time points. Mice without oral administration of Cy5.5-modified PDA NDs were defined as pre. For the ex vivo imaging, Cy5.5-modified PDA NDs were orally administered to mice at first. Then, mice were sacrificed at any expected time point. Major organs including heart, liver, spleen, lung, kidney, stomach, small intestine, and colon were individually collected and imaged under an in vivo imaging system. To re-confirm the above bio-distribution results of PDA NDs, IBD mice was also developed after the oral administration of DSS (2.5%) for one week, which could be considered as a mild IBD model in mice. Similar investigation for the bio-distribution of PDA NDs after oral administration were carried out according to our above design.

Mucoadhesive property of PDA NDs. Cy5.5-modified PDA NDs were orally administrated to healthy and IBD nude mice, respectively. 12 h later, mice were sacrificed and colons were collected. Then, colons were imaged under an in vivo imaging system after repeated rinsing with 0.9% NaCl.

Endocytosis capacity of Caco-2 cells towards PDA NDs and PDA NPs. Caco-2 cells were seeded in 6-well plates at a density of 5×10^5 cells/well and incubated for 48 h. After medium was replaced with fresh medium containing Cy5.5-modified nanoparticles (50 μg/mL), cells were incubated for another 6 h. Subsequently, cells were treated with DAPI and FITC-labeled phalloidin in the dark for 0.5 h. After rinsing with serum-free medium, fluorescence images were captured with the assistance of a confocal microscope.

Protective effects of PDA NDs on Caco-2 cells after Rosup exposure. Caco-2 cells were seeded in a 24-well plate at a density of 5×10^3 cells/well. After the cellular attachment, medium was replaced with fresh medium containing FBS (10%) and PDA NDs (100 μg/mL). 4 h later, Rosup was added into the above system. At the end of experiment with a co-incubation period of 6 h, cells were treated with MTT for another 4 h, which was followed by the addition of DMSO to dissolve the formazan crystals. A microplate reader was utilized to measure the absorbance values of above wells at 490 nm.

Intracellular ROS scavenging assay. ROS Assay Kit was utilized to quantify the intracellular ROS after different treatments. Caco-2 cells were seeded in a 6-well plate with a density of 5×10^5 cells/well. After the cellular attachment, the above cells were randomly divided into 4 groups including control, PDA NDs, Rosup, and Rosup+PDA NDs. The incubation periods of Rosup and PDA NDs in the above groups were defined as 6 h. At the end of the incubation, DCFH-DA working solution was added into each well and incubated in the dark for another 0.5 h. After rinsing with serum-free medium, intracellular ROS levels were evaluated using a confocal microscope and a flow cytometer.

Live/dead staining of cells after antioxidant treatment. Caco-2 cells were seeded in a 6-well plate at a density of 5×10^5 cells/well and incubated for 48 h. After medium was replaced with fresh medium containing PDA NDs (100 μg/mL), cells were incubated for another 4 h. Then, cells were incubated with Rosup (50 μg/mL) for 6 h. Cells without any treatment were defined as the control group. At the end of the whole experimental period, cells were stained with Calcein AM/PI working solution in the dark for 0.5 h. After rinsing with serum-free medium, fluorescence images were captured with the help of a confocal microscope.

β-galactosidase staining. Typically, Caco-2 cells were cultured in a 6-well plate and incubated for 48 h. After receiving different treatments, cells were fixed with 4% paraformaldehyde and incubated with β-galactosidase staining solution at 37°C overnight. Then, the expression of β-galactosidase in various groups was recorded under an optical microscope. The degrees of β-galactosidase expression were quantified using an ImageJ software.

DNA damage assay of Caco-2 cells. Caco-2 cells were seeded into a 6-well plate and incubated for

48 h. After the cellular attachment, PDA NDs (100 μg/mL) were added into the above wells. 4 h later, cells were treated with Rosup for another 6 h. At the end of the whole experiment, cells were fixed with 4% paraformaldehyde. Then, degreased milk was utilized to block samples for 0.5 h. After washing with 0.9% NaCl, cells were co-incubated with anti-γH2AX antibody. Finally, cells were treated with anti-rabbit AlexaFluor-488 and DAPI. Fluorescence images were captured under a confocal microscope. The degree of DNA damage was quantified using an ImageJ software.

Anti-apoptotic performance of PDA NDs. Caco-2 cells were seeded in a 6-well plate and incubated for 48 h. After medium was replaced with fresh medium containing PDA NDs (100 μg/mL), cells were incubated for another 4 h. Then, cells were incubated with Rosup for another 6 h. Cells without any treatment were defined as the control group. At the end of the whole experiment, we collected the treated cells, followed the guideline of Annexin V-FITC/PI Apoptosis Detection Kit, as well as analyzed the anti-apoptotic effect of PDA NDs using a flow cytometer.

Evaluation of intracellular oxidative damage biomarkers. Typically, Caco-2 cells were firstly incubated with PDA NDs for 4 h. Then, Rosup was added into the above medium. 6 h later, intracellular oxidative damage biomarkers were quantified with the help of MDA Assay Kit and SOD Assay Kit, respectively.

Establishment of DSS-induced acute colitis and the corresponding treatment. C57BL/6J mice after adaptive feeding were randomly divided into 6 groups including control, PDA NPs, PDA NDs, DSS, DSS+PDA NPs, and DSS+PDA NDs. Subsequently, the drinking water in the groups of DSS, DSS+PDA NPs, and DSS+PDA NDs was replaced with DSS (3%). A week later, mice in the groups of DSS+PDA NPs and DSS+PDA NDs were orally administered with PDA NPs (20 mg/kg) and PDA NDs (20 mg/kg), respectively. Meanwhile, mice in the group of PDA NPs and PDA NDs were orally administered with the same dose of PDA NPs and PDA NDs. Mice in the group of control were orally administered with 0.9% NaCl. After 3 d of continuous oral administration, changes in body weight, stool viscosity, hematochezia, mortality, and physical condition were recorded and analyzed. At the end of the whole experiment, mice in the above 6 groups were sacrificed, and colons were collected

for the further measurement of colon length, histological assessment, and evaluations of oxidative damage biomarkers. Moreover, mice in the above 6 groups after cardiac perfusion were treated with DCFH-DA, and the achieved colons were imaged under an in vivo imaging system. Last but not least, 5-ASA was selected as the classical positive drug to explore the practical therapeutic effect of our PDA NDs. Similar experiments including the development of DSS-induced acute colitis and the corresponding treatment were well performed. During the experiment, mice were orally administered with 5-ASA at 50 mg/kg per day.

Microbiome analysis. A pea-sized fecal sample was taken from the intestine of each mouse, packaged, and subjected to the microbiome analysis. After extracting the total DNA from the samples, primers were designed according to the conserved regions, and sequencing adapters were added to the ends of primers. The products were amplified by PCR, purified, quantified, and normalized to create the sequencing libraries. The constructed libraries were first subjected to the quality inspection, and the qualified libraries were sequenced using the Illumina NovaSeq 6000. The resulting raw sequence reads were transformed into original sequencing reads (Sequenced Reads) through base calling analysis and stored in FASTQ format files. The Raw Reads obtained from sequencing were filtered using a Trimmomatic v0.33 software. To obtain Clean Reads without primer sequences, the primer sequences were identified and removed with the help of a cutadapt 1.9.1 software. Usearch v10 software was utilized to align the Clean Reads of each sample, and then the data was filtered according to the length range for different regions. The DADA2 method in QIIME2 2020.6 software was utilized to perform denoise and remove chimeric sequences, which could result in the acquisition of the final high-quality data (non-chimeric reads). Alpha-diversity was measured using the Chao1 and Shannon indices. PCoA analysis was utilized to compare the differences between different groups.

Transcriptomics analysis. At the end of the above experiment, colons from normal mice, the mice with DSS-induced colitis, and the IBD mice after PDA NDs-assisted treatment were collected and stored at -80°C. Total RNA was extracted using a Total RNA Extractor (Trizol) Kit (Sangon, China) based on the manufacturer’s protocol, and treated with RNase-free DNase I to remove genomic DNA

contamination. RNA integrity was evaluated with a 1.0% agarose gel. The concentrations and purities of RNA were measured using a NanoPhotometer® spectrophotometer (IMPLEN, USA) and a Qubit® 2.0 Flurometer (Invitrogen), respectively. Following the reverse transcription of RNA (150-200 bp in length) to cDNA and the amplification of primed cDNA, RNA-seq was performed on a NovaSeq sequencer (Illumina, USA). The resulting clean reads were mapped to the reference genome using HISAT2 (version 2.0) with default parameters. RSeQC (version 2.6.1) was utilized to statistics the alignment results. The homogeneity distribution and the genome structure were checked with the help of Qualimap (version 2.2.1). BEDTools (version 2.26.0) was utilized to perform the statistical analysis of the gene coverage ratio. The data were processed and visualized using R v4.4.1 and various R packages, including "prcomp" for generating the principal component plot. To achieve a volcano plot, differentially expressed genes (DEGs) were performed using the R package "Limma" ($|Log_2FC| > 1$; $p < 0.05$). Heatmaps were utilized to visualize the genes with significant variability. To explore the biological function and enriched pathways, Gene Ontology (GO) and Kyoto Encyclopedia of Genes and Genomes (KEGG) enrichment analyses were conducted using the R package "clusterProfiler". The *p* values were calculated using the hypergeometric test and adjusted for the multiple testing using the Benjamini-Hochberg correction.

Establishment of DSS-induced chronic colitis and the corresponding treatment. C57BL/6J mice after adaptive feeding were randomly divided into 6 groups including control, PDA NPs, PDA NDs, DSS, DSS+PDA NPs, and DSS+PDA NDs. Subsequently, we replaced the drinking water in the groups of DSS, DSS+PDA NPs and DSS+PDA NDs with DSS (2.5%). A week later, mice in the groups of PDA NPs, PDA NDs, DSS+PDA NPs, and DSS+PDA NDs were orally administered with PDA NPs and PDA NDs for continuous 8 d. It was worth noting that DSS was continuously utilized within the above half a month. Detailed grouping information and specific treatment protocol were similar to those utilized in the acute colitis model. During the experiment, changes in body weight, stool viscosity, hematochezia, and physical condition were recorded and analyzed. At the end of the whole experiment, mice in the above 6 groups were sacrificed, and colons were collected for the

further measurement of colon length and histological analysis. In addition, 5-ASA was utilized as the typical positive drug to explore the practical therapeutic effect of our PDA NDs. Similar experiments including the development of DSS-induced chronic colitis and the corresponding treatment were well performed. During the experiment, mice were orally administered with 5-ASA at 50 mg/kg per day.

Establishment of TNBS-induced colitis and the corresponding treatment. C57BL/6J mice after adaptive feeding were randomly divided into 6 groups including control, PDA NPs, PDA NDs, TNBS, TNBS+PDA NPs, and TNBS+PDA NDs. Prior to the establishment of TNBS-induced model of colitis, mice were deprived of food and kept in water for 1 d. Mice in the groups of TNBS, TNBS+PDA NPs, and TNBS+PDA NDs were treated with a TNBS/ethanol enema (2%, w/v). Then, mice in the groups of PDA NPs, PDA NDs, TNBS+PDA NPs, and TNBS+PDA NDs were orally administered with PDA NPs (20 mg/kg) and PDA NDs (20 mg/kg), respectively. Meanwhile, mice in the groups of control and TNBS were administered with 0.9% NaCl. 4 d later, changes in body weight, stool viscosity, hematochezia, physical condition, food consumption, and mortality were recorded using a protocol similar to that for DSS-induced colitis.

DHE staining of colonic tissues. Firstly, collected colonic tissues were made into the frozen slides. Secondly, the frozen slides were restored to room temperature, eliminated obvious liquid, and marked the objective tissue with liquid blocker pen. Auto-fluorescence quenching reagent was added to avoid the unnecessary auto-fluorescence of tissues. Thirdly, DHE was added to the marked the area after washing under running water, and the slides were incubated in the dark at 37℃. Fourthly, the slides were treated with DAPI. Finally, the slides were covered with anti-fade mounting medium and fluorescence images were collected under a confocal microscope.

Histopathological analysis. For the hematoxylin-eosin (H&E)-stained histological evaluation, main organs including heart, liver, spleen, lung, kidney, stomach, small intestine, and colon were carefully harvested at the end of the whole experiment, fixed with 4% paraformaldehyde, and embedded in paraffin for staining. Moreover, colons achieved from different IBD models were fixed in 4% paraformaldehyde and made into paraffin sections for the staining of H&E, TUNEL, toluidine blue

(TB), and zonula occludens-1 (ZO-1), respectively.

Statistical analysis. Statistical analysis was carried out using OriginPro 2022 and GraphPad Prism 9. Significant differences were analyzed using unpaired *t*-test for two groups and one-way analysis of variance (ANOVA) for multiple group comparisons. All data in this study were expressed as mean ± standard deviation (SD) from at least 3 independent experiments. The p value less than 0.05 was considered statistically significant.

2. Supplementary figures and tables

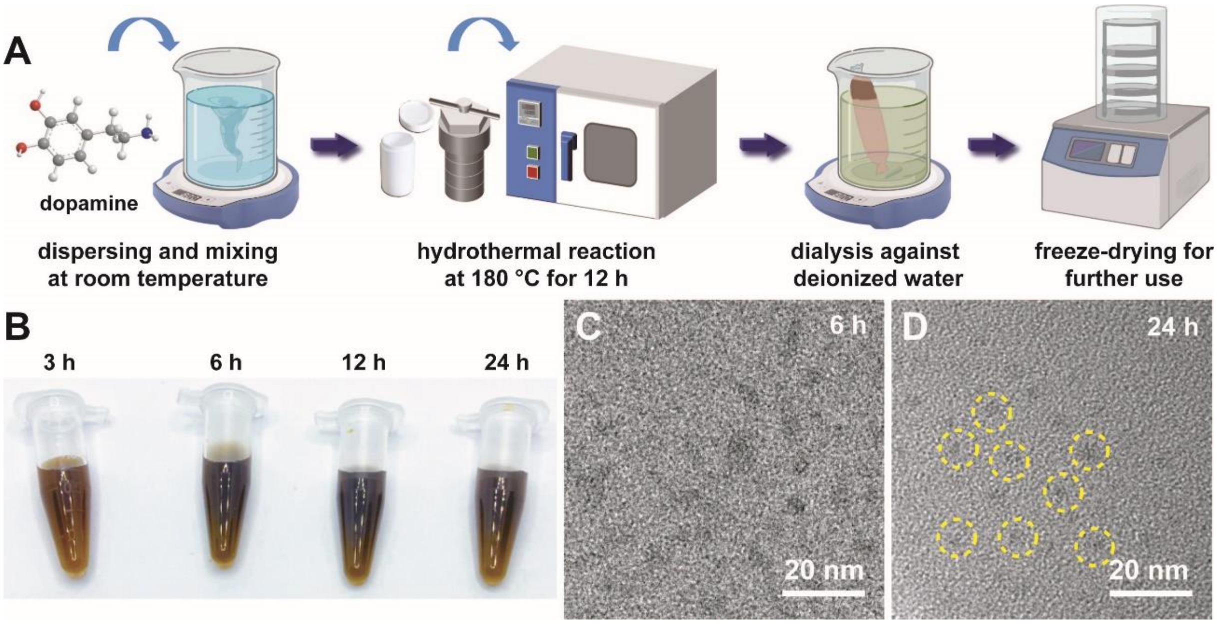

Figure S1. Schematic illustration for the synthesis of PDA NDs (A). Photos of dispersions containing PDA NDs prepared with different reaction periods (B). TEM images of PDA NDs prepared with different reaction periods including 6 h (C) and 24 h (D).

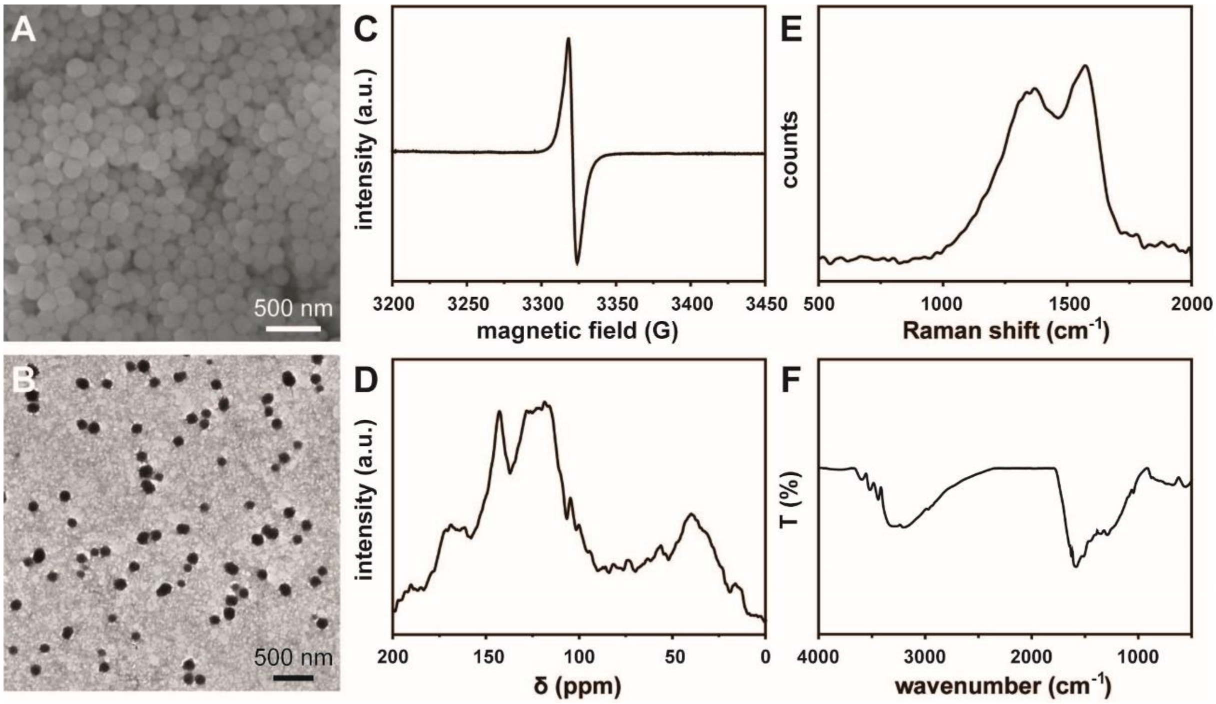

Figure S2. SEM image (A), TEM image (B), ESR spectrum (C), ^{13}C NMR spectrum (D), Raman spectrum (E), and FT-IR spectrum (F) of PDA NPs.

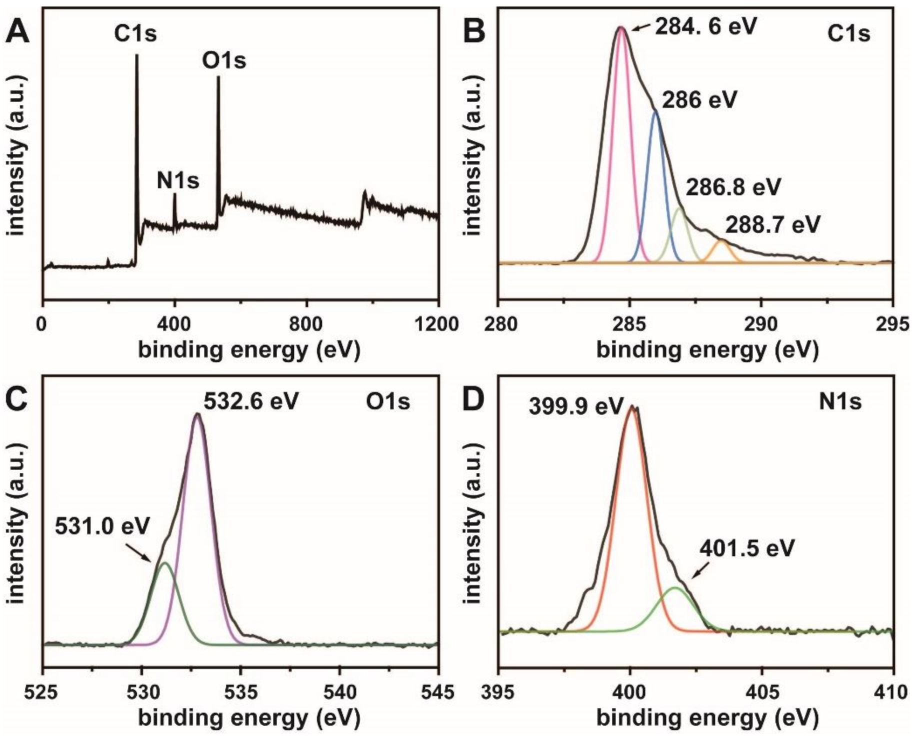

Figure S3. XPS survey spectrum (A), as well as high-resolution C 1s (B), O 1s (C), and N 1s (D) spectra of PDA NDs.

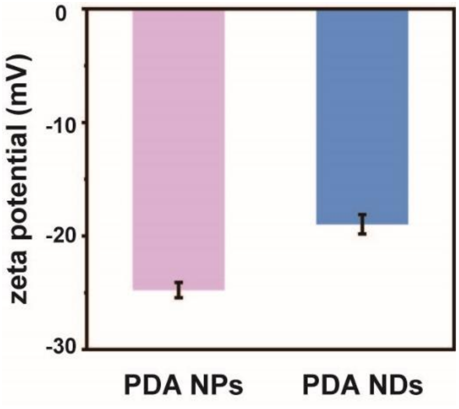

Figure S4. Zeta potentials of PDA NPs and PDA NDs. Data are presented as mean ± SD (n = 3).

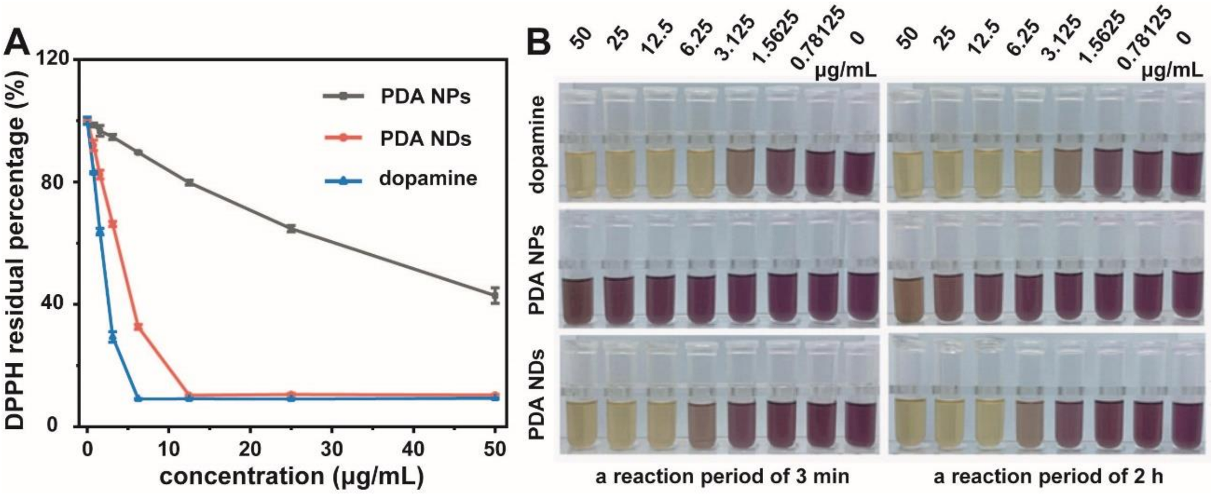

Figure S5. DPPH residual percentage after a 2 h treatment by PDA NDs, PDA NPs, and dopamine (A), as well as the corresponding photos with different reaction periods (B). Data in (A) are presented as mean ± SD (*n* = 3).

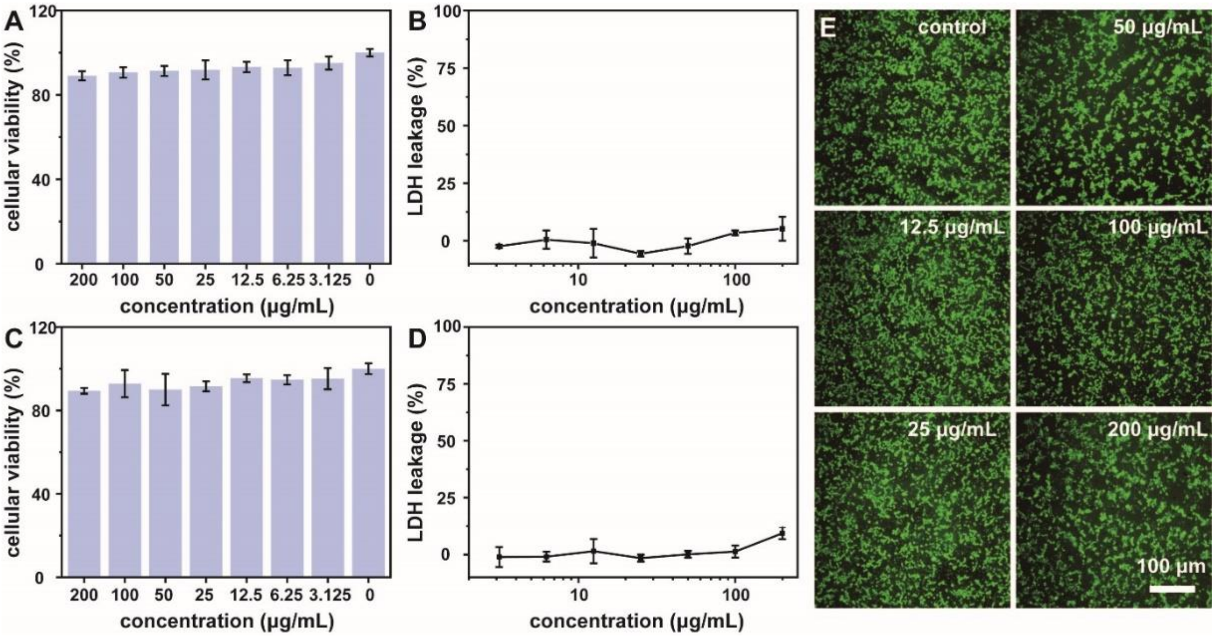

Figure S6. Cytotoxicity of PDA NDs on Caco-2 cells based on MTT assay (A) and LDH leakage assay (B). Cytotoxicity of PDA NDs on RAW 264.7 cells based on MTT assay (C), LDH leakage assay (D), and visible live/dead staining (E). Data in (A) and (C) are presented as mean ± SD (*n* = 5). Data in (B) and (D) are presented as mean ± SD (*n* = 3).

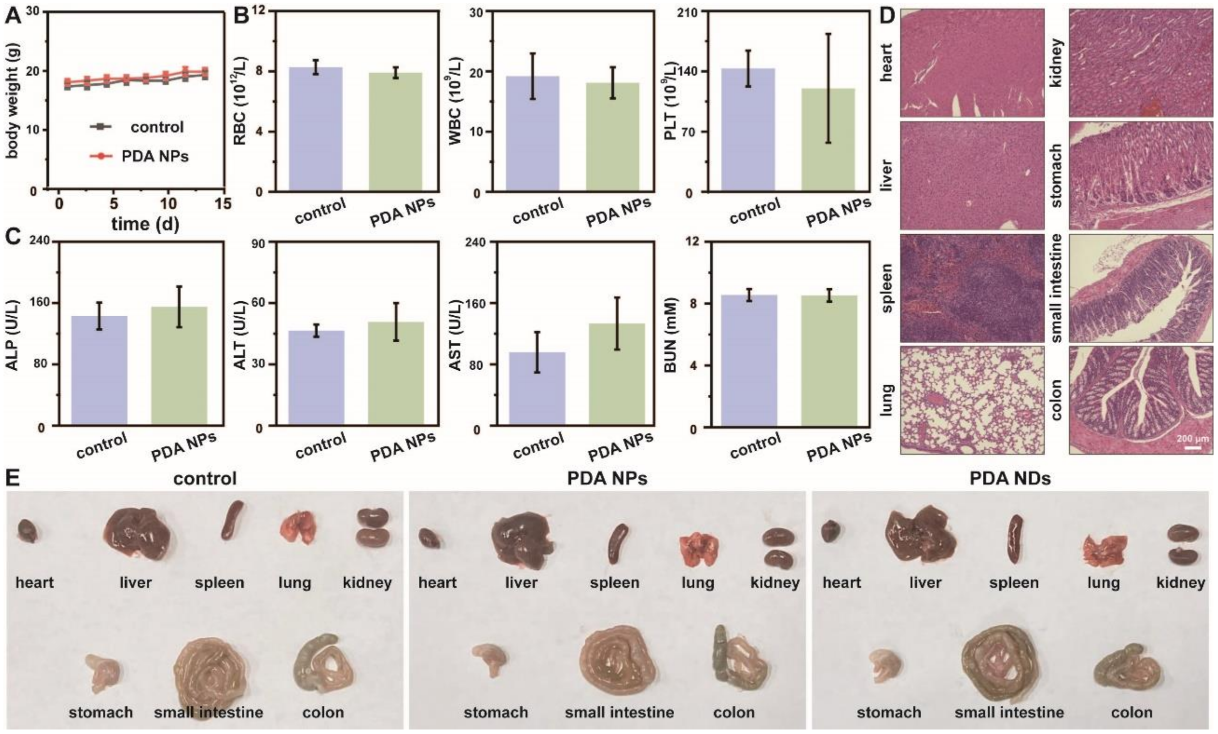

Figure S7. Changes of body weight (A), hematological index (B), blood biochemical index (C), and histopathological analysis of main organs (D) from mice after oral administration of PDA NPs. Photos of main organs from mice 14 d after different treatments (E). Data in (A) are presented as mean ± SD (n = 5). Data in (B) and (C) are presented as mean ± SD (n = 3).

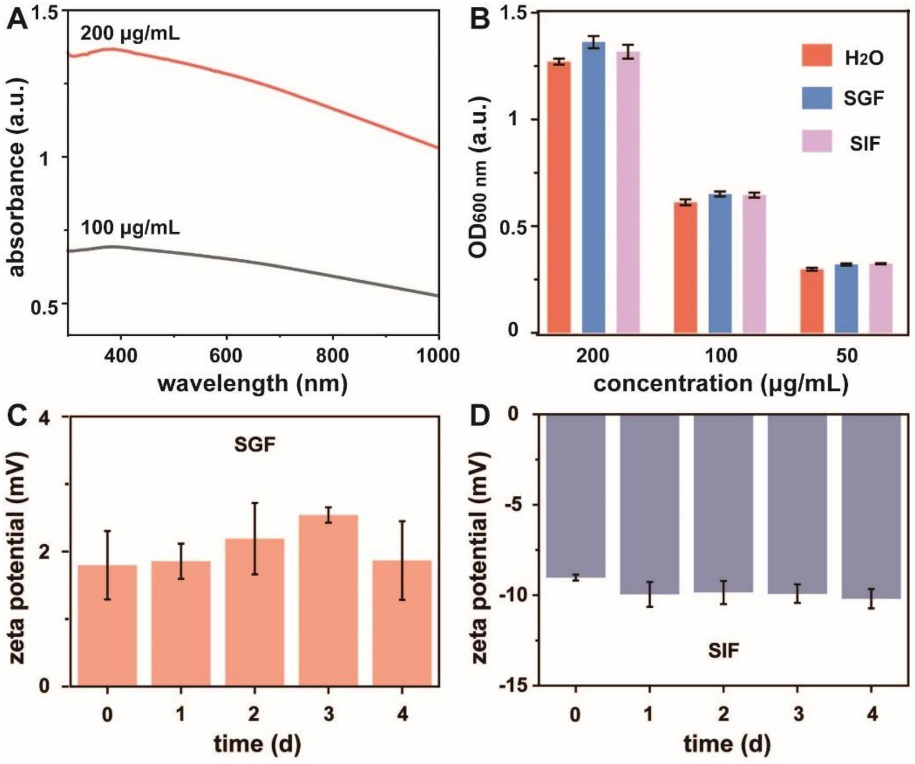

Figure S8. UV-vis spectra of PDA NDs with different concentrations (A) and the corresponding $OD_{600\ nm}$ under different conditions (B). Time-dependent zeta potential changes of PDA NDs in SGF (C) and SIF (D). Data in (B), (C), and (D) are presented as mean ± SD (*n* = 3).

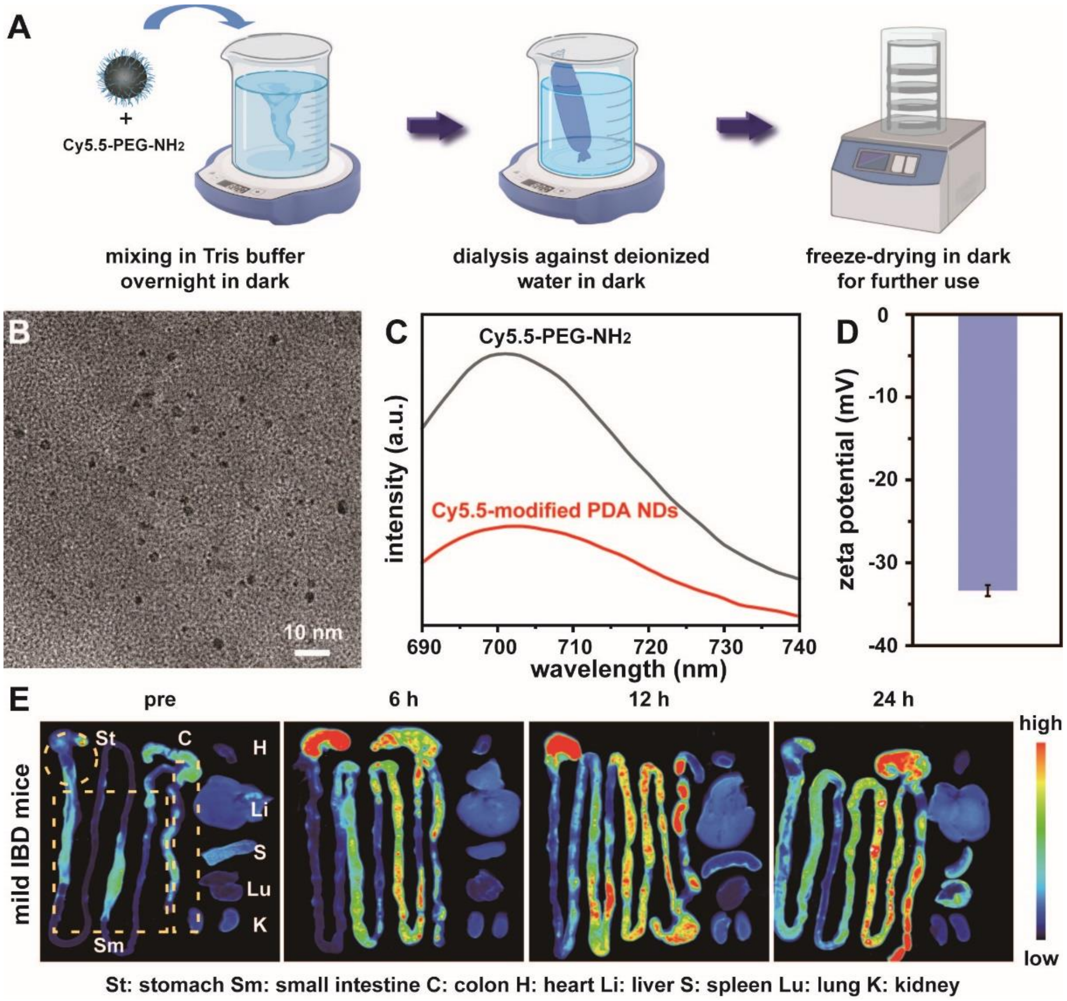

Figure S9. Schematic illustration of the synthesis of Cy5.5-modified PDA NDs (A). TEM image (B), fluorescent spectrum (C), and zeta potential of Cy5.5-modified PDA NDs (D). Time-dependent ex vivo fluorescence imaging of mild IBD mice after oral administration of Cy5.5-modified PDA NDs (E). The above IBD mice were developed by oral administration of DSS with a concentration of 2.5%. Data in (D) are presented as mean ± SD (*n* = 3).

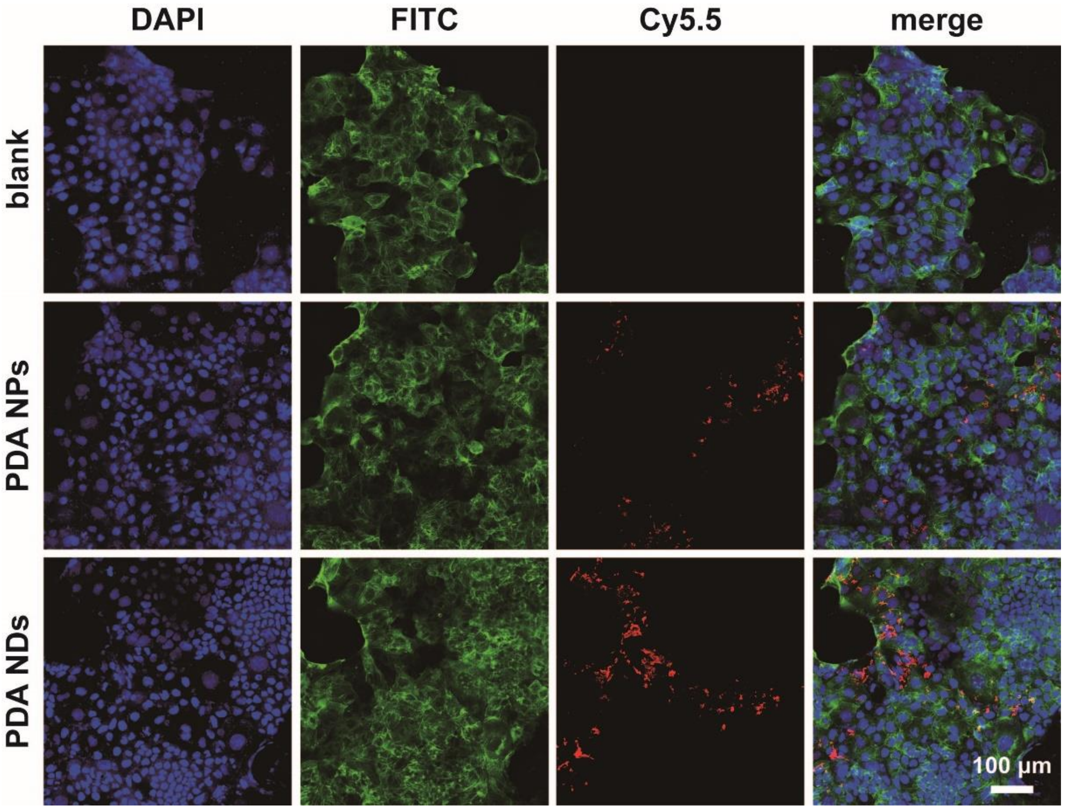

Figure S10. Endocytosis performances of Caco-2 cells towards PDA NDs and PDA NPs. Nucleus and cytoskeletons of Caco-2 cells were labelled with DAPI and FITC while PDA NDs and PDA NPs were labelled with Cy5.5.

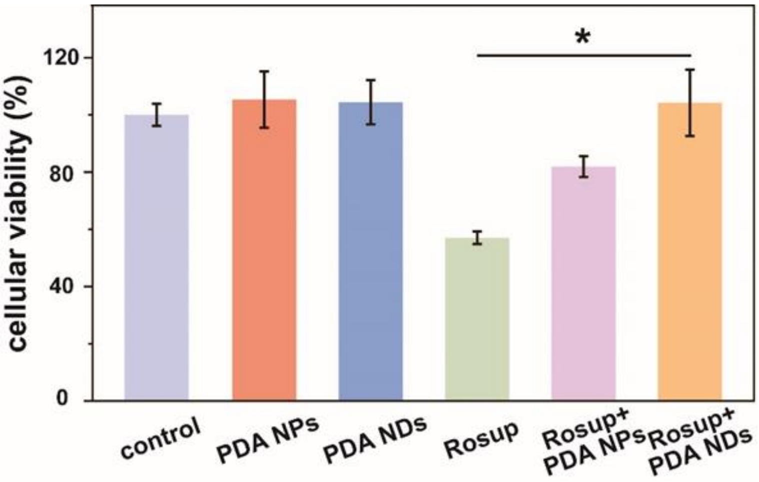

Figure S11. Protective effects of PDA NDs and PDA NPs on Rosup-treated Caco-2 cells. Data are presented as mean ± SD (n = 3). Statistical significance is calculated using one-way ANOVA with multiple comparison tests. *p < 0.05.

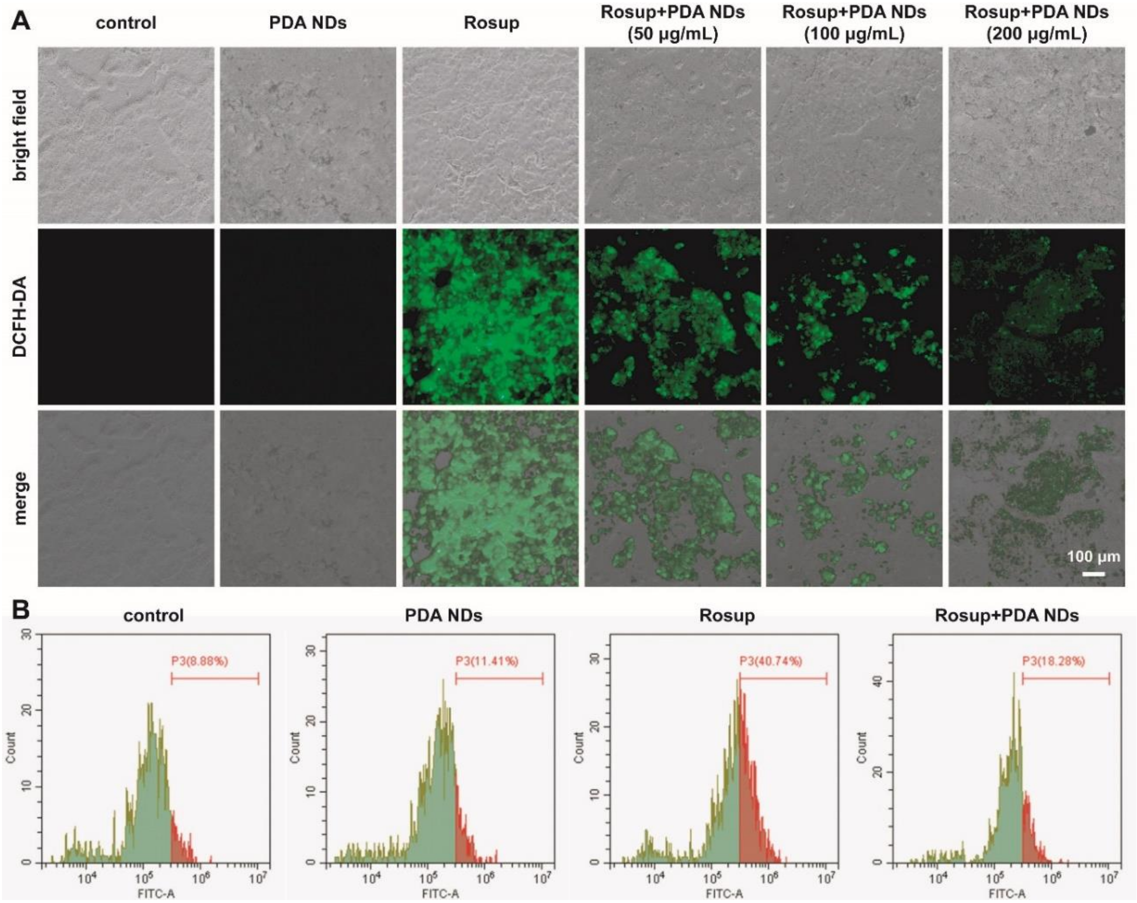

Figure S12. Fluorescence images of Caco-2 cells after different treatments indicated by DCFH-DA (A). Intracellular ROS quantification of Caco-2 cells after different treatments according to flow cytometry (B).

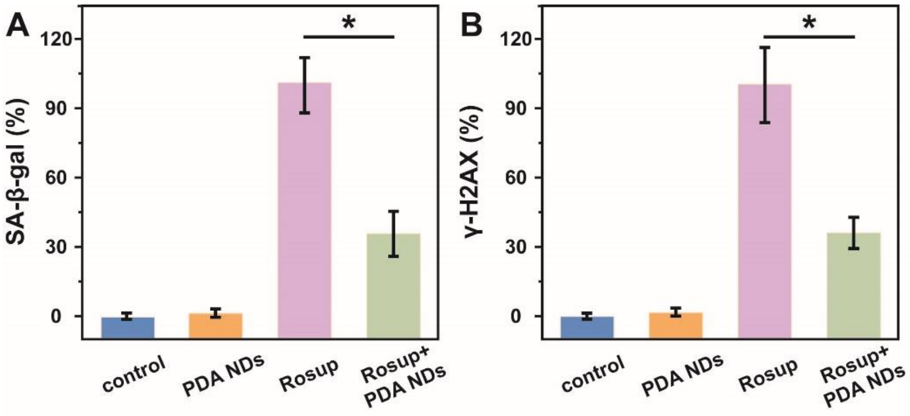

Figure S13. Intracellular SA-β-gal (A) and γ-H2AX (B) quantification of Caco-2 cells after different treatments. Data in (A) and (B) are presented as mean ± SD (n = 3). Statistical significance is calculated using unpaired t-test. *$p < 0.05$.

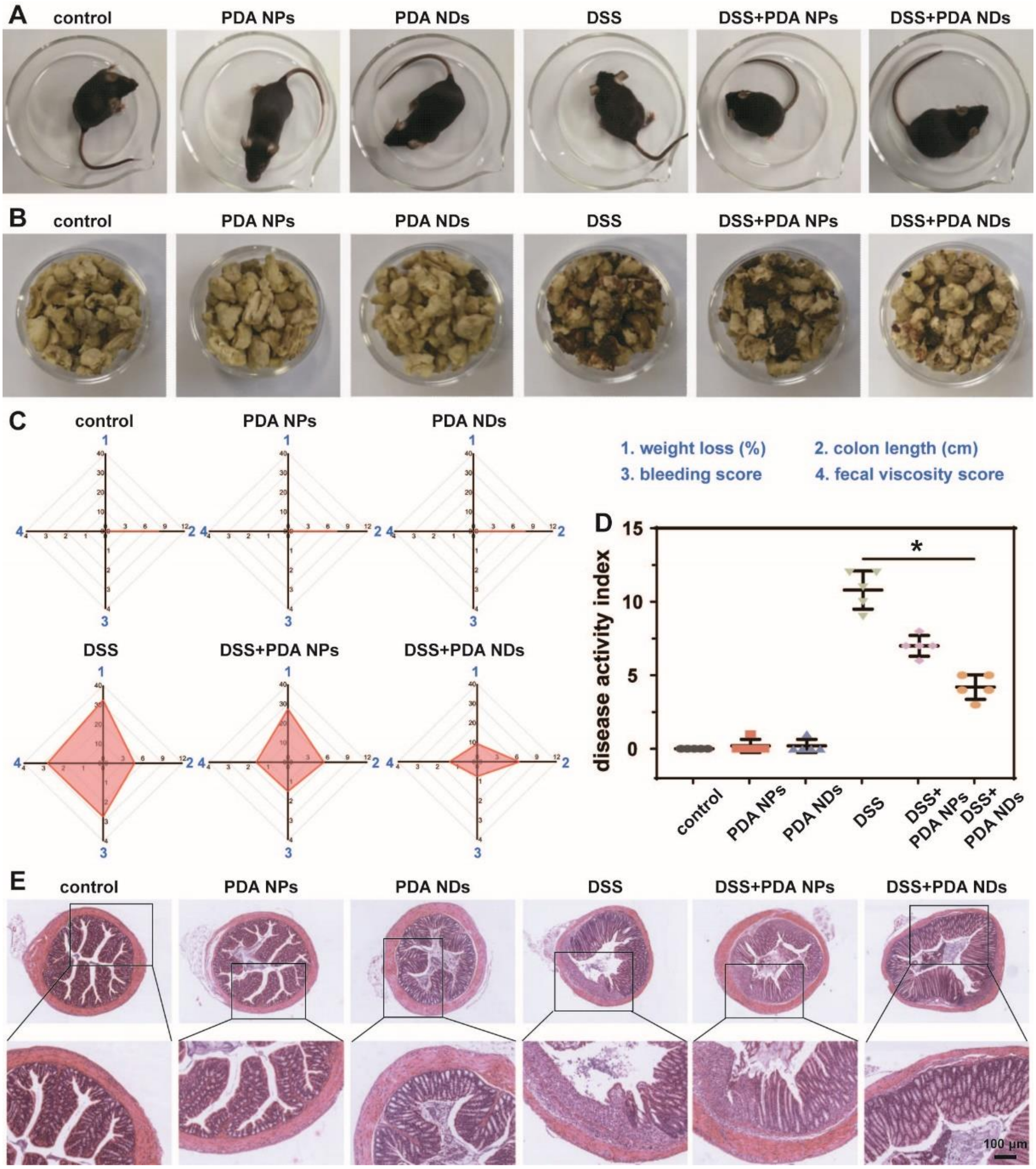

Figure S14. Photos of mouse behavior (A) and the corresponding bedding (B) in different groups. Colonic damage scores of mice after different treatments (C). Disease activity index of mice based on the above individual damage score (D). Enlarged H&E staining images of the colonic tissue specimens from mice after different treatments (E). Data in (D) are presented as mean ± SD (n = 5). Statistical significance in (D) is calculated using one-way ANOVA with multiple comparison tests. *$p < 0.05$.

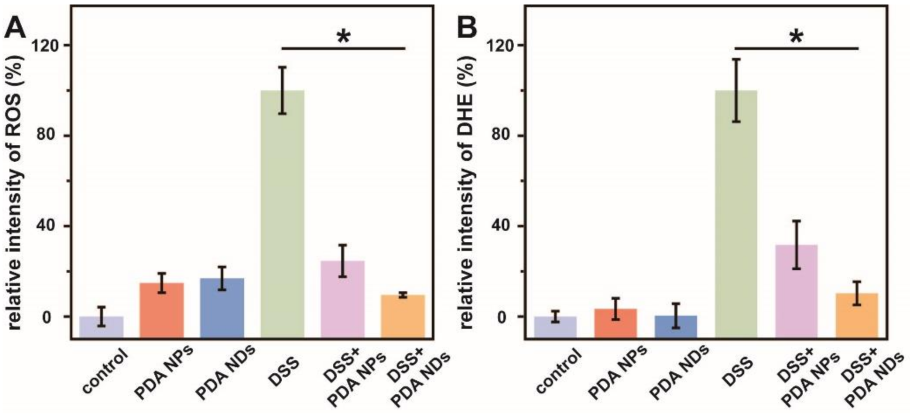

Figure S15. Quantification of the relative intensity of ROS (A) and DHE (B) in mice after different treatments. Data are presented as mean ± SD (n = 3). Statistical significance is calculated using one-way ANOVA with multiple comparison tests. *$p < 0.05$.

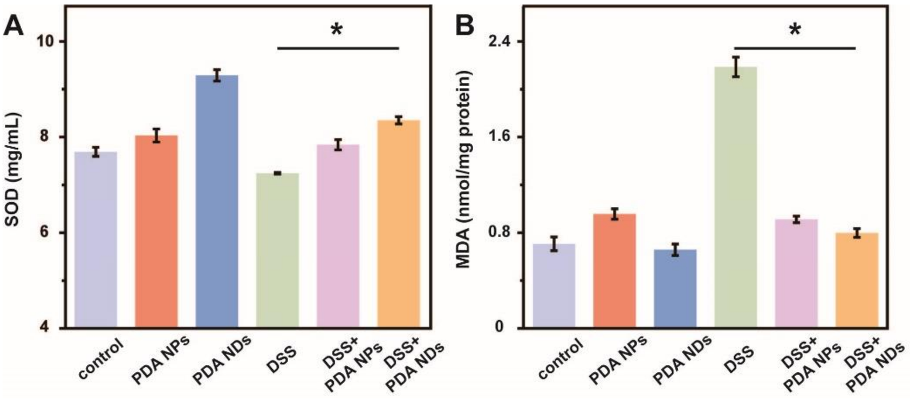

Figure S16. Quantification of SOD (A) and MDA (B) in the colonic tissues from different mice. Data in (A) and (B) are presented as mean ± SD (n = 3). Statistical significance is calculated using one-way ANOVA with multiple comparison tests. *$p < 0.05$.

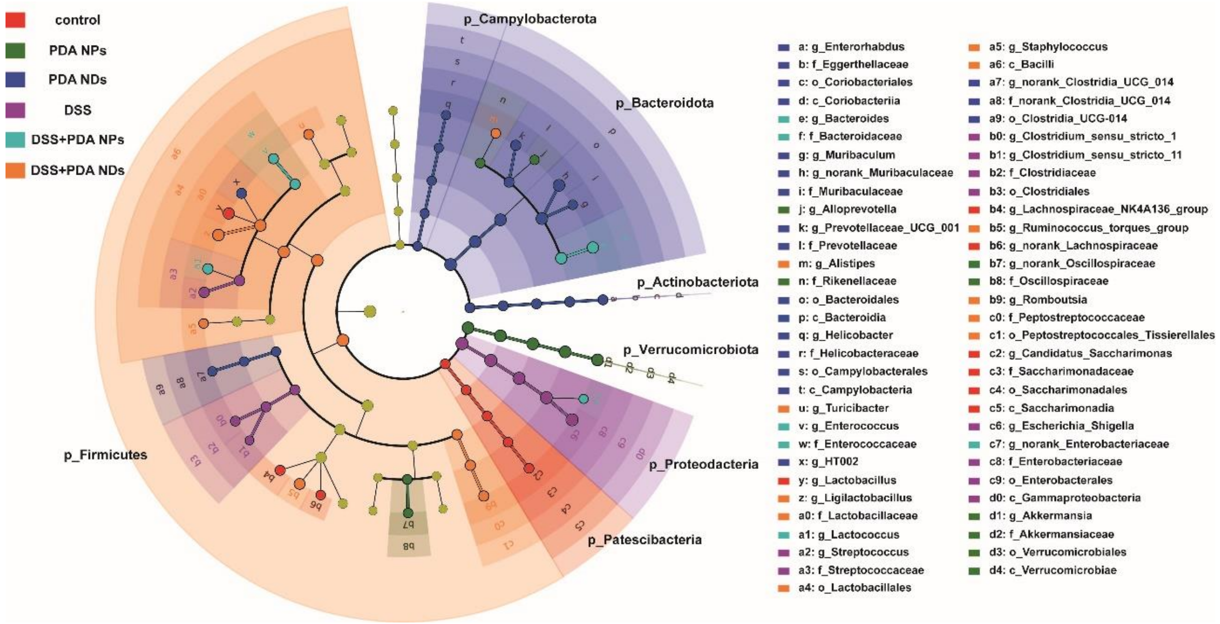

Figure S17. Taxonomic cladogram generated from LEfSe analysis at the genus level indicating communities or species with significantly varying effects after different treatments.

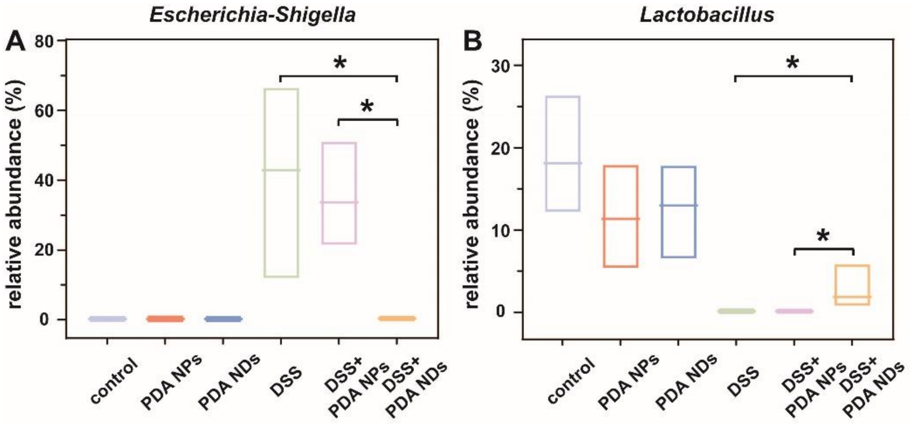

Figure S18. Relative abundance of selected taxa at the genus level from mice after different treatments. Data in (A) and (B) are presented as mean ± SD (n = 5). Statistical significance is calculated using unpaired t-test. *$p < 0.05$.

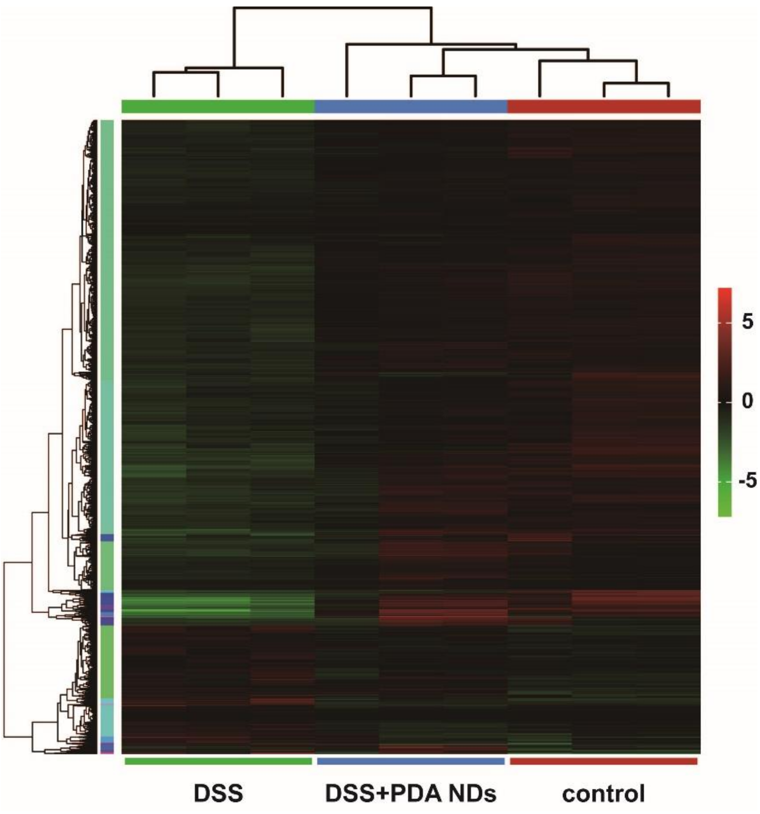

Figure S19. Heatmap demonstrating differentially expressed transcripts among colonic tissues from mice in different groups.

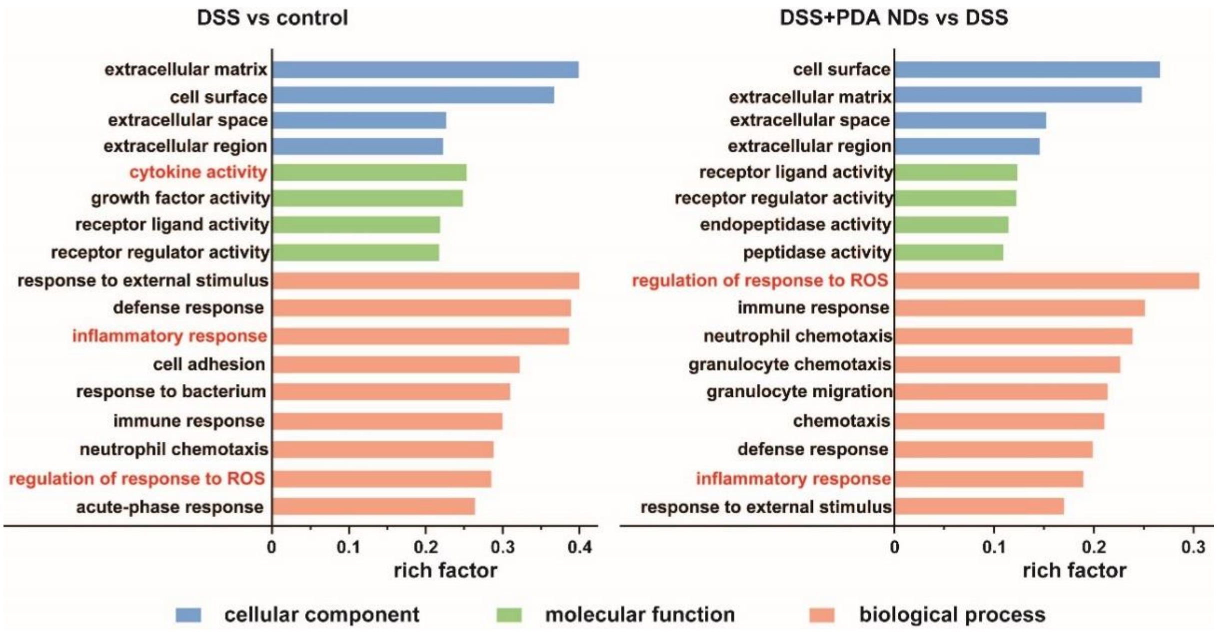

Figure S20. GO analysis of DEGs in the colonic tissues from mice in different groups.

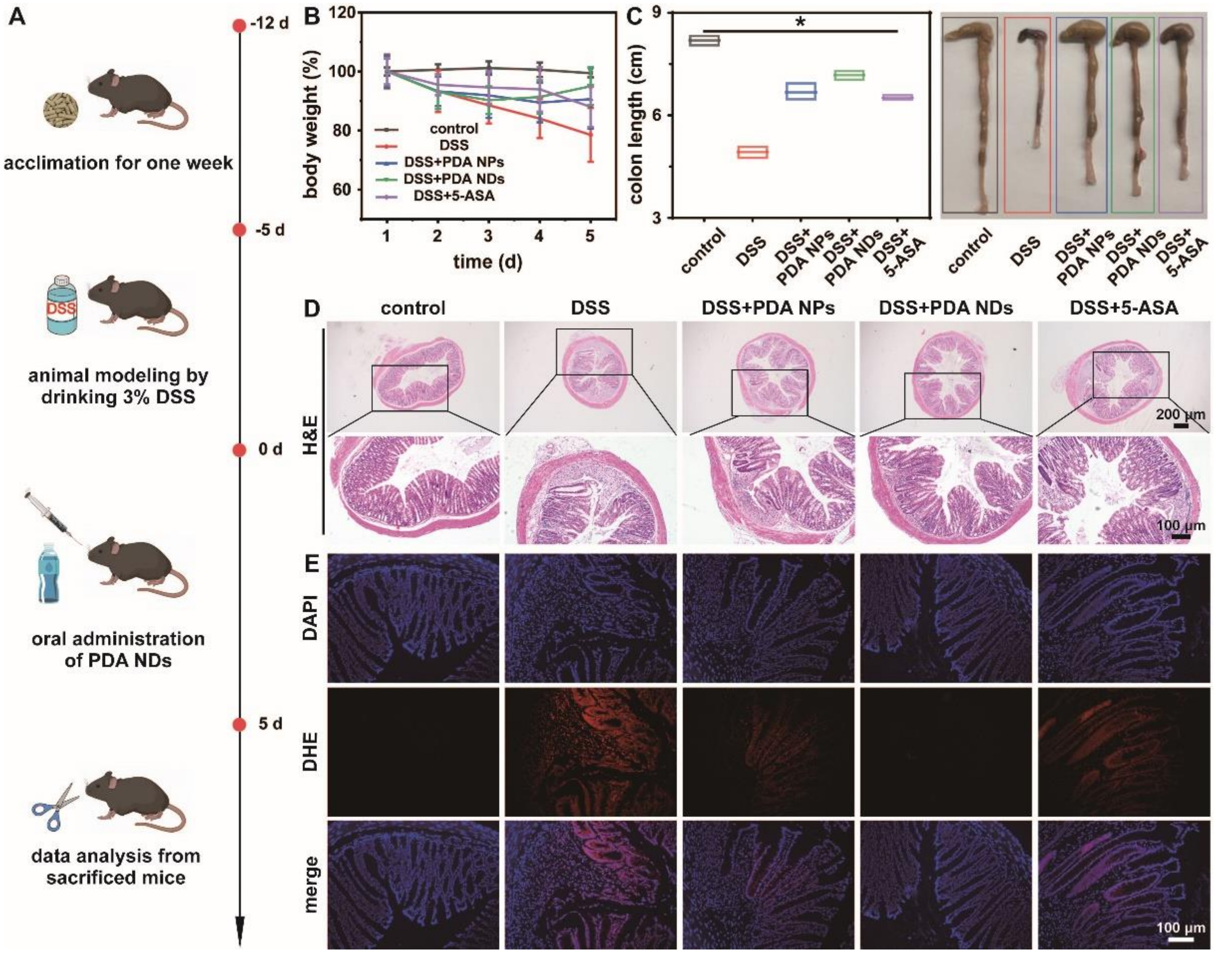

Figure S21. Schematic illustration of the establishment of DSS-induced acute colitis and the corresponding treatment in the presence of positive drug (A). Changes in body weight of mice during the treatment (B). Colon length and the corresponding photos of colonic tissues obtained from mice after different treatments (C). H&E staining images of the colonic tissue specimens from mice after different treatments (D). Fluorescence images of ROS in different colonic tissue specimens, indicated by DHE (E). Data in (B) and (C) are presented as mean ± SD (n = 5). Statistical significance in (C) is calculated using one-way ANOVA with multiple comparison tests. *p < 0.05.

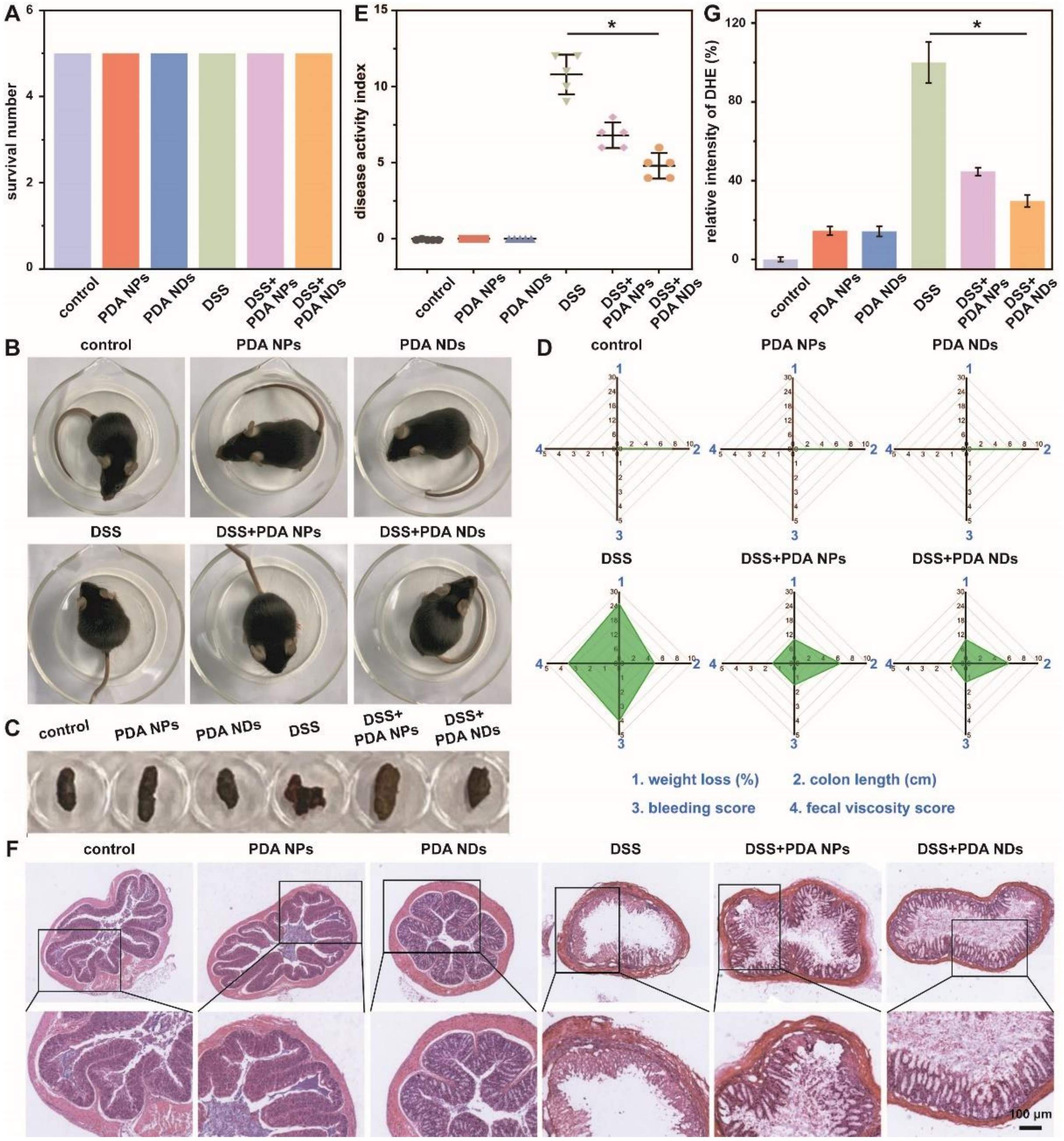

Figure S22. Survival number of mice after different treatments (A). Photos of mouse behavior (B) and feces (C) in different groups. Colonic damage scores of mice receiving different treatments (D). Disease activity index of mice according to the above individual damage score (E). Enlarged H&E staining images of the colonic tissue specimens from mice after different treatments (F). Relative intensity of DHE in mice after different treatments (G). Data in (E) are presented as mean ± SD (n = 5). Data in (G) are presented as mean ± SD (n = 3). Statistical significance in (E) and (G) is calculated using one-way ANOVA with multiple comparison tests. *$p < 0.05$.

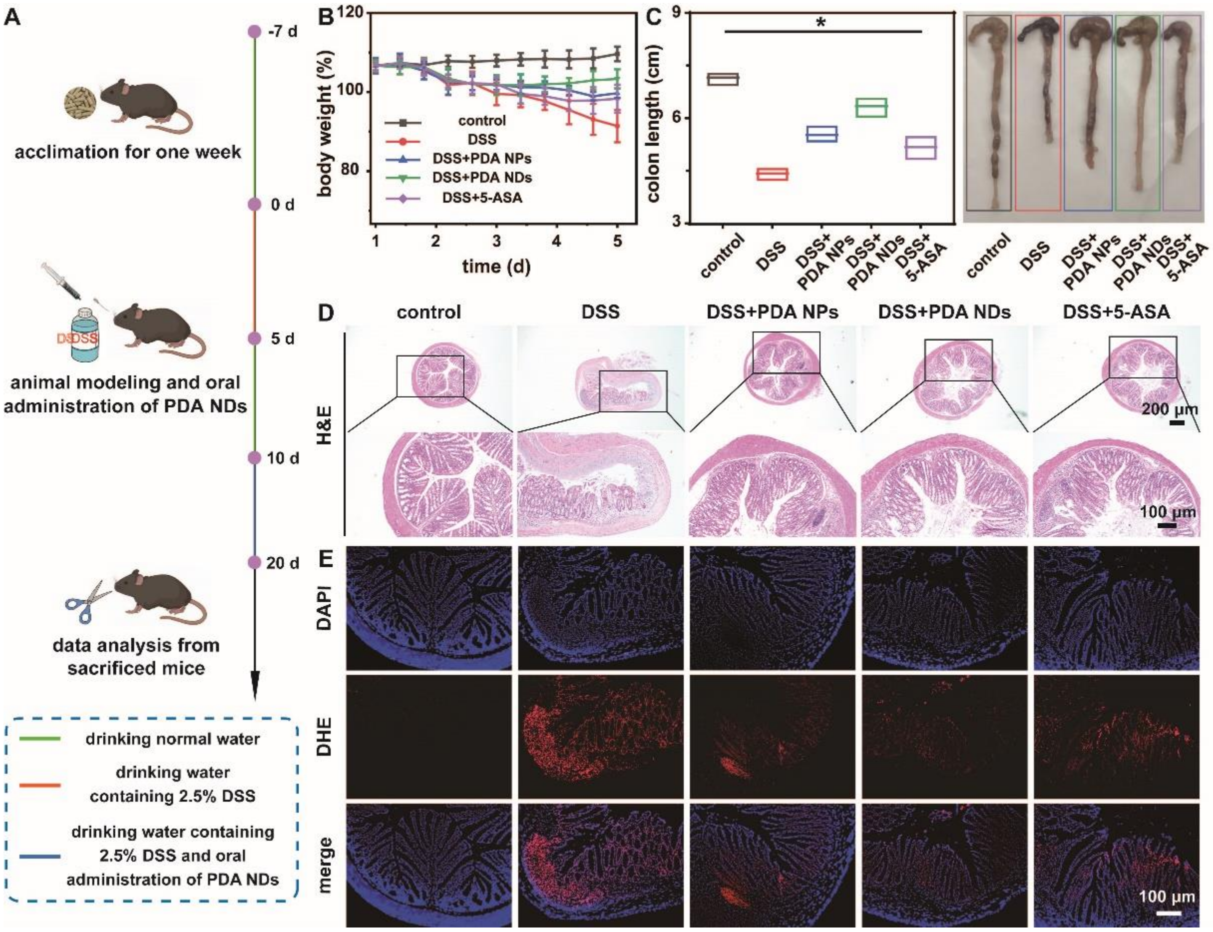

Figure S23. Schematic illustration of the establishment of DSS-induced chronic colitis and the corresponding treatment in the presence of positive drug (A). Changes in body weight of mice during the treatment (B). Colon length and the corresponding photos of colonic tissues obtained from mice after different treatments (C). H&E staining images of the colonic tissue specimens from mice after different treatments (D). Fluorescence images of ROS in different colonic tissue specimens, indicated by DHE (E). Data in (B) and (C) are presented as mean ± SD (n = 5). Statistical significance in (C) is calculated using one-way ANOVA with multiple comparison tests. *$p < 0.05$.

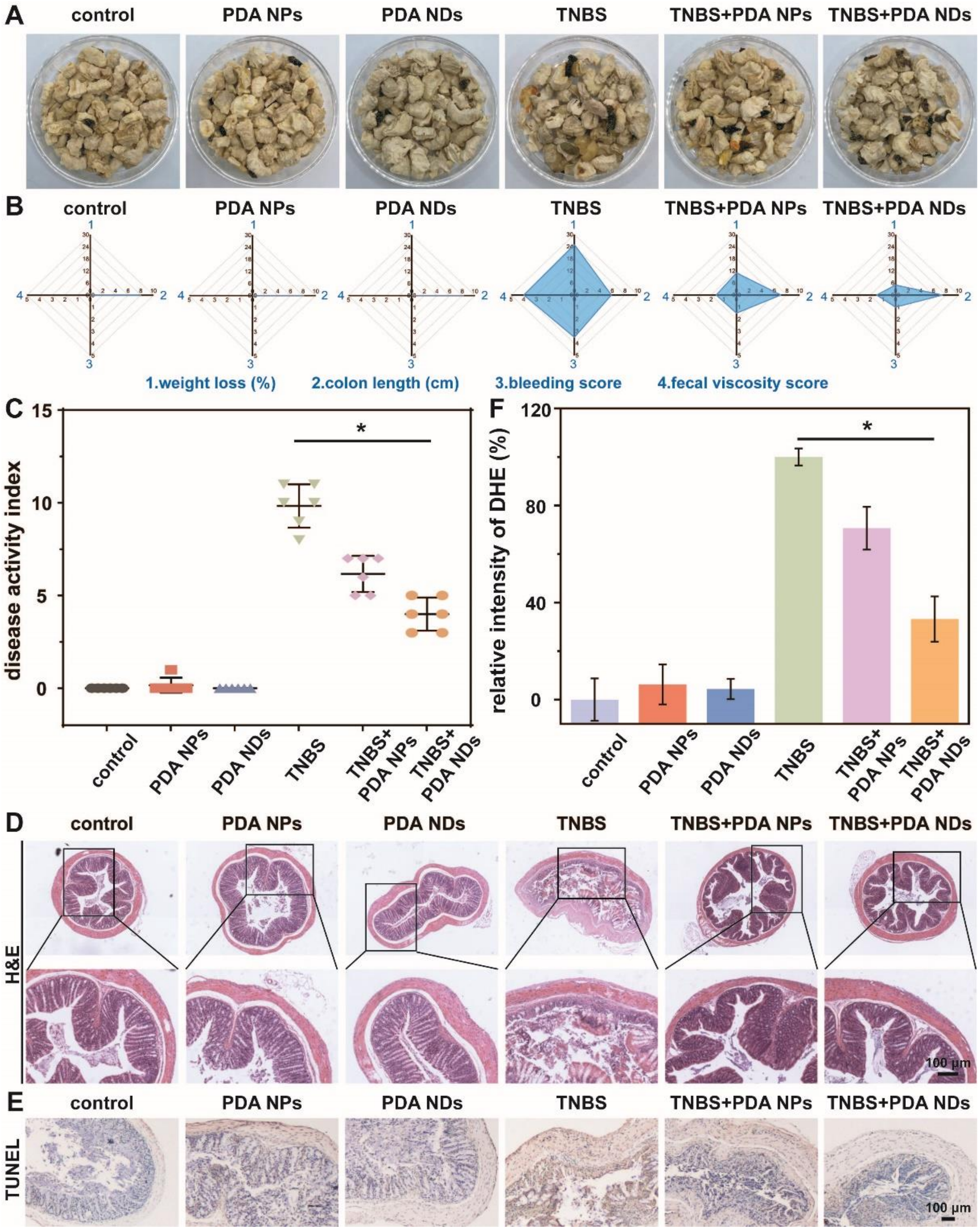

Figure S24. Photos of bedding in different groups (A). Colonic damage scores of mice after different treatments (B). Disease activity index of mice according to the above individual damage score (C). Enlarged H&E staining images of the colonic tissue specimens from mice after different treatments (D). TUNEL assay of colonic tissues from mice in different groups (E). Relative intensity of DHE in mice after different treatments (F). Data in (C) are presented as mean ± SD ($n = 5$). Data in (F) are presented as mean ± SD ($n = 3$). Statistical significance in (C) and (F) is calculated using one-way ANOVA with multiple comparison tests. *$p < 0.05$.

Table S1. Average hemolysis rates 2 h after the incubation with PDA NDs.

concentration (μg/mL)	hemolysis (%)
0.9% NaCl	0
H_2O	100
25	0.448
50	0.418
100	0.567
200	0.806
400	1.493

Table S2. Average clotting time of PT, TT, and APTT, and average fibrinogen (FIB) content 3 h after the incubation with PDA NDs.

concentration (μg/mL)	PT time (s)	TT time (s)	APTT time (s)	FIB content (g/L)
0	9.53	11.37	35.6	3.433
25	10.1	11.53	36.1	3.195
50	9.8	11.2	29	3.516
100	10.2	11.43	31.53	3.322
200	10.27	11.63	32.8	3.467
400	11	11.8	32.33	3.293

Table S3. Urinalysis of C57BL/6J mice in the groups of control, PDA NPs and PDA NDs.

Group	WBC (cells/μL)	protein (g/L)	pH value	blood (cells/μL)	specific gravity	bilirubin (μmol/L)
control	-	-	5.5	-	1.030	-
PDA NPs	-	-	5.0	-	1.030	-
PDA NDs	-	-	6.0	-	1.025	-

Table S4. Scoring system for the status of mice with DSS-induced acute colitis.

score	weight loss (%)	colon length loss (%)	stool viscosity	hematochezia
0	<5	<5	normal	normal
1	5-10	5-20	loose	perceptible
2	20-30	20-35	mild	slight
3	>30	>35	liquid	gross

1. Weight loss percentage = (weight at the beginning of experiment - weight at the end of experiment)/weight at the beginning of experiment × 100%.

2. When the mouse died during the experiment, the body weight at death was the mouse weight at the end of experiment.

3. Colon length loss percentage = (colon length at the beginning of experiment - colon length at the end of experiment)/colon length at the beginning of experiment × 100%.

Table S5. Scoring system for the status of mice with DSS-induced chronic colitis.

score	weight loss (%)	colon length loss (%)	stool viscosity	hematochezia
0	<5	<5	normal	normal
1	5-10	5-10	loose	mild
2	10-15	10-20	sticky	moderate
3	15-20	20-30	mushy	serious

Table S6. Scoring system for the status of mice with TNBS-induced colitis.

score	weight loss (%)	colon length loss (%)	stool viscosity	hematochezia ($OD_{652\ nm}$)
0	<5	<5	normal	<0.15
1	5-10	5-15	loose	0.15-0.3
2	10-15	15-25	sticky	0.3-0.45
3	15-20	25-30	mushy	0.45-0.6

Because the hematochezia degree of TNBS-induced CD model was very mild, the differences between groups could not be found by naked eyes. Therefore, colorimetric analysis was utilized to evaluate the level of hematochezia in mice. In common, ferroporphyrin, also named as heme, in red blood cells with intrinsic peroxidase like activity could catalyze the oxidation of TMB towards the generation of colored oxTMB in the presence of H_2O_2. Typically, mouse feces in each group were crushed and added into the TMB-contained buffer containing TMB (1 mM) and H_2O_2 (50 mM) at first. Subsequently, the hematochezia degrees of different samples were evaluated according to the absorbance values of dispersions at 652 nm.
